# Quinoline-functionalized graphene oxide for enhanced cadmium removal: synthesis, characterization, and mechanistic insights

**DOI:** 10.1039/d6ra00200e

**Published:** 2026-03-13

**Authors:** Huda Ammar, Mohamed F. Kamel, Ahmed M. Masoud, Entsar H. Taha, Adel A. El-Zahhar, Majed M. Alghamdi, Mohamed H. Taha

**Affiliations:** a Nuclear Materials Authority P. O. Box 530, El Maddi Cairo Egypt ammar.huda@yahoo.com chemmaso010@hotmail.com; b Department of Plant Protection, Faculty of Agriculture, Ain Shams University Cairo Egypt; c Department of Chemistry, Faculty of Science, King Khalid University P.O.Box 9004 Abha 61413 Saudi Arabia

## Abstract

Cadmium contamination in aquatic systems poses serious environmental and human health risks, driving the need for efficient, selective, and regenerable adsorbents. Herein, graphene oxide (GO) was functionalized with 8-hydroxyquinoline (GQ) and 8-hydroxyquinoline-5-sulfonic acid (GQS) *via* carbodiimide-mediated coupling to introduce strong chelating N,O- and N,O/S-donor sites for enhanced Cd(ii) removal. Comprehensive characterization using X-ray diffraction (XRD), Fourier-transform infrared spectroscopy (FTIR), scanning electron microscopy coupled with energy-dispersive X-ray spectroscopy (SEM-EDS), Brunauer–Emmett–Teller (BET), dynamic light scattering (DLS), and zeta-potential measurements confirmed successful ligand grafting, surface functionalization, and a controlled reduction in surface area. Batch adsorption experiments revealed optimal Cd(ii) uptake at pH 6 and a sorbent dose of 2.0 g L^−1^, with rapid adsorption kinetics well described by the pseudo-second-order model (*R*^2^ > 0.998). Equilibrium data fitted the Langmuir and Sips isotherms, yielding maximum adsorption capacities of 39.9, 32.9, and 26.6 mg g^−1^ for GQ, GQS, and GO, respectively, reflecting the increasing density and accessibility of chelation sites. Thermodynamic analysis (Δ*H*° < 0, Δ*G*° < 0) indicated a spontaneous, exothermic adsorption process governed primarily by chelation-driven inner-sphere complexation and interfacial dehydration. The sorbents exhibited excellent regenerability, achieving 93.6% Cd(ii) desorption using 1.0 M HCl. Importantly, treatment of a real industrial raffinate resulted in 94.2% Cd(ii) removal with minimal co-ion interference, confirming high selectivity under realistic conditions. Overall, this study demonstrates that quinoline-functionalized graphene oxide provides a simple, tunable, and reusable platform for high-performance cadmium remediation in complex aqueous matrices.

## Abstract 2

Cadmium contamination in aquatic systems poses serious environmental and human health risks, driving the need for efficient, selective, and regenerable adsorbents.

## Graphical abstract

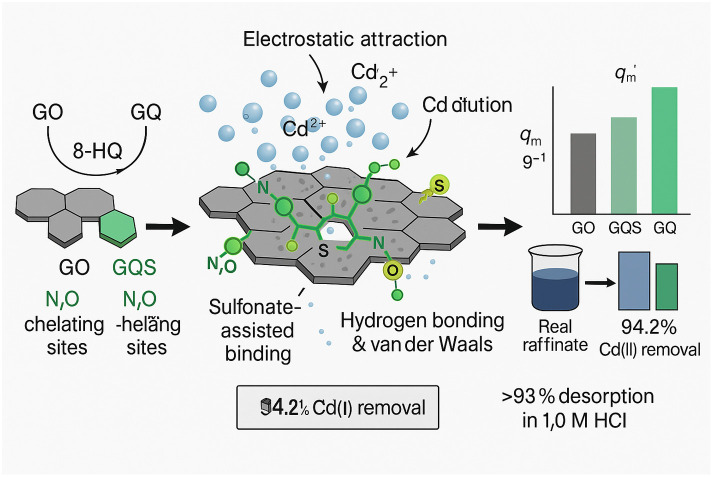

## 1 Introduction

Heavy-metal contamination in aquatic systems represents a critical global environmental challenge due to the persistence, bioaccumulation, and high toxicity of metal ions released from industrial discharges, mining activities, electroplating, pigment production, phosphate processing, and related operations.^1–3^ Among toxic metals, cadmium (Cd(ii)) is particularly hazardous because of its high solubility, long biological half-life, and tendency to accumulate in vital organs, leading to renal dysfunction, skeletal damage, cardiovascular toxicity, neurological impairment, and carcinogenic effects even at trace levels.^1–3^ Its widespread presence in industrial effluents results in concentrations that often exceed regulatory limits, prompting strict maximum permissible levels such as the WHO guideline of <0.003 mg L^−1^ in drinking water.^3,4^ These concerns underscore the urgent need for efficient, selective, and sustainable technologies for Cd(ii) abatement.

Several conventional treatment methods—including chemical precipitation,^5^ ion exchange,^6^ membrane filtration,^7^ electrochemical processes,^8^ and solvent extraction^9^—have been explored for Cd(ii) remediation. However, these techniques generally suffer from high operational costs, low selectivity, membrane fouling, incomplete removal at low concentrations, and problematic sludge generation.^10,11^ Adsorption has therefore attracted substantial attention as a practical alternative due to its low cost, simplicity, rapid operation, and strong capability for removing heavy metals from dilute solutions.^12–14^ Nonetheless, traditional adsorbents such as activated carbon,^15^ clays,^16^ biochars,^17^ and zeolites^18^ often exhibit limited capacities, slow kinetics, or poor regeneration performance—particularly under competitive or variable pH conditions.^12–14^ These shortcomings have driven the development of advanced, chemically engineered adsorbents with tailored surface functionalities and stronger affinity toward toxic metal ions.

Graphene oxide (GO) has become one of the most promising sorbents for Cd(ii) due to its high surface area, hydrophilicity, and rich oxygenated functional groups capable of electrostatic attraction and complexation.^19–23^ However, GO often exhibits moderate affinity toward Cd(ii), as its oxygen-based sites form mainly monodentate, weakly binding complexes. This limitation has fueled extensive efforts to modify GO through a variety of structural, chemical, and hybridization strategies. Among these, amine-functionalized GO materials, for example, introduce abundant electron-donating groups that substantially enhance metal–ligand coordination and improve uptake of Cd(ii), Cu(ii), Pb(ii), and Cr(vi).^24^ A GO/paper hybrid prepared from waste fibers exhibited promising adsorption of Pb(ii), Ni(ii), and Cd(ii) while maintaining strong regeneration performance.^25^ Magnetic GO composites—including GO-tryptophan (MGO-Trp),^26^ α-Fe_2_O_3_/GO,^27^ few-layered magnetic graphene oxide (FLMGO),^28^ and PAN = Fe_3_O_4_@GO^29^ frameworks—offer enhanced adsorption capacities, faster diffusion pathways, and facile magnetic recovery, thereby improving operational practicality and recyclability. Other advanced approaches include cyclodextrin-GO hybrids, where host–guest interactions and hierarchical porosity enable exceptional Cd(ii) capacities,^30,31^ as well as GO modified with sulfur- and nitrogen-rich ligands, which promote strong multidentate binding with soft metal ions.^32,33^ Collectively, these studies highlight that GO adsorption performance is governed not solely by surface area, but more critically by the nature, density, and accessibility of surface functional groups and hybrid components.

Recent research trends have increasingly shifted from maximizing adsorption capacity alone toward improving selectivity, mechanistic understanding, and applicability under realistic wastewater conditions. Emphasis has been placed on graphene oxide derivatives and hybrid architectures—such as magnetic, biopolymer-supported, metal-oxide-decorated, and sulfonated graphene-based systems—that strengthen inner-sphere coordination with Cd(ii) while maintaining performance near neutral pH and in the presence of competing ions.^23,27,30,33,34^ In parallel, regeneration efficiency, stability during reuse, and synthetic simplicity have emerged as key criteria for the practical deployment of graphene-based adsorbents in wastewater treatment processes.^30,34^ Despite these advances, clear structure–property–performance relationships that directly link ligand chemistry and surface functionality to adsorption energetics, selectivity, and real-sample behavior remain insufficiently established, particularly for chelation-driven Cd(ii) removal systems.

Within this context, chelation-based functionalization using aromatic heterocycles has emerged as a chemically rational and effective strategy for strengthening metal–ligand interactions. In particular, 8-hydroxyquinoline (8-HQ) and its derivatives have attracted substantial interest because of their strong *N*,*O*-bidentate chelating ability, rigid aromatic backbone, and high stability constants with numerous metal ions.^20–22^ When covalently immobilized or strongly anchored on GO, the quinoline nitrogen and phenolic oxygen act cooperatively to form robust inner-sphere complexes, markedly enhancing binding affinity and imparting selectivity toward borderline and soft Lewis acids such as Cd(ii). The sulfonated analogue, 8-hydroxyquinoline-5-sulfonic acid (HQS), introduces an additional –SO_3_H/–SO_3_^−^ group, further increasing surface acidity, hydrophilicity, and electrostatic attraction, thereby improving Cd(ii) adsorption efficiency.^21,22^ Despite their demonstrated promise, systematic, side-by-side comparisons of GO, 8-HQ-functionalized GO (GQ), and HQS-functionalized GO (GQS) under identical experimental conditions remain limited. Moreover, the mechanistic effects of quinoline-based functionalization on GO's structure, surface charge, coordination environment, and adsorption energetics are not yet fully understood. In addition, many high-capacity graphene-based Cd(ii) sorbents reported to date rely on multistep synthesis routes—such as inorganic oxide loading, polymer grafting, or magnetic architectures—which can complicate scalability and obscure the intrinsic role of ligand chemistry in governing adsorption behavior.^35–40^ In contrast, ligand-driven functionalization strategies enable adsorption performance to be tuned primarily through coordination chemistry, allowing clearer mechanistic interpretation and structure–property–performance correlations under realistic aqueous conditions.

Motivated by these considerations, the present study reports the synthesis, comprehensive characterization, and adsorption performance of three sorbents: pristine graphene oxide (GO), GO functionalized with 8-hydroxyquinoline (GQ), and GO functionalized with 8-hydroxyquinoline-5-sulfonic acid (GQS). The materials were characterized using X-ray diffraction, Fourier-transform infrared spectroscopy, scanning electron microscopy coupled with energy-dispersive X-ray spectroscopy, Brunauer–Emmett–Teller/Barrett–Joyner–Halenda textural analysis, dynamic light scattering, and zeta-potential measurements. Batch adsorption experiments were conducted to evaluate the effects of solution pH, sorbent dosage, contact time, initial Cd(ii) concentration, and temperature, with the resulting data analyzed using kinetic, isotherm, and thermodynamic models. Regeneration experiments and real-wastewater application tests were further performed to assess reusability, selectivity, and practical applicability. Overall, this work provides mechanistic insight into how quinoline-based functionalization modulates the physicochemical properties of GO and enhances its affinity, selectivity, and regenerability for Cd(ii) removal. Notably, this ligand-driven strategy offers a comparatively simple and tunable route to upgrading GO performance without reliance on inorganic nanoparticles or polymeric scaffolds, while maintaining high removal efficiency near neutral pH and demonstrating selective Cd(ii) uptake in a real industrial raffinate matrix.

## 2 Experimental

### 2.1 Materials

Natural graphite powder, potassium permanganate (KMnO_4_), phosphoric acid (H_3_PO_4_), and hydrogen peroxide (H_2_O_2_, 30%) used for GO synthesis were obtained from Merck (Germany). 8-Hydroxyquinoline (8-HQ, C_9_H_7_NO) and 8-hydroxyquinoline-5-sulfonic acid (HQS, C_9_H_7_NO_4_S) were purchased from Sigma–Aldrich (USA) and used as received. Cadmium sulfate (CdSO_4_·*x*H_2_O, ≥99%) was also obtained from Sigma–Aldrich and used to prepare Cd(ii) stock and working solutions. Hydrochloric acid (HCl), nitric acid (HNO_3_), sulfuric acid (H_2_SO_4_), sodium hydroxide (NaOH), and ethanol (CH_3_CH_2_OH) were supplied by Loba Chemie (India). A 1000 mg L^−1^ Cd(ii) stock solution was prepared by dissolving an accurately weighed amount of CdSO_4_ in deionized water and was freshly diluted to the desired concentrations prior to each experiment. The pH of the working solutions was adjusted using 0.1–1.0 M HCl or NaOH and monitored with a calibrated pH meter. Deionized water (resistivity ≥ 18.2 MΩ cm) was used throughout all synthesis, adsorption, and washing steps.

### 2.2 Synthesis of sorbents

Graphene oxide (GO) was used as the parent carbonaceous scaffold for preparing the two functionalized sorbents, GQ and GQS. The functionalization procedures were designed to introduce chelating N,O-donor groups (8-hydroxyquinoline) or N,O/S-bearing groups (8-hydroxyquinoline-5-sulfonic acid) onto the GO surface through a mild activation–coupling strategy, thereby increasing the density of metal-binding sites while preserving the lamellar structure of GO.

#### 2.2.1 Preparation of GO

Graphene oxide (GO) was synthesized using a modified Hummers' method.^19,20^ In this procedure, 1.5 g of graphite powder and 9.0 g of potassium permanganate (KMnO_4_) were slowly added to a mixed acid solution containing concentrated H_3_PO_4_ and H_2_SO_4_ under continuous stirring for 12 h. After completion, the reaction mixture was allowed to cool to room temperature and was then gradually poured onto 200 mL of ice containing 2 mL of 30% H_2_O_2_. The color change from brown to yellow confirmed the successful oxidation of graphite. The resulting suspension was centrifuged at 5000 rpm, and the solid product was washed sequentially with deionized water, dilute HCl, and ethanol to remove residual acids and metal ions. Finally, the purified GO powder was dried under vacuum at room temperature for 24 h.

#### 2.2.2 Synthesis of GO functionalized with 8-hydroxyquinoline (GQ)

The 8-hydroxyquinoline-functionalized graphene oxide (GQ) composite was prepared using a solution-based ligand loading method.^21,22^ First, a saturated solution of 8-hydroxyquinoline (8-HQ) in water–ethanol was sonicated for 2 hours to ensure dissolution, then filtered (0.45 µm) to yield a clear solution. To this, 1.0 g of graphene oxide (GO) was added, and the mixture was continuously stirred at room temperature for 48 hours to promote interaction between the 8-HQ and the GO surface. The resulting solid GQ composite was then isolated, washed repeatedly with deionized water until colorless to remove unbound 8-HQ, and finally dried in an oven at 60 °C overnight.

#### 2.2.3 Synthesis of GO functionalized with 8-hydroxyquinoline-5-Sulfonic acid (GQS)

The 8-hydroxyquinoline-5-sulfonic acid-functionalized graphene oxide (GQS) composite was synthesized using a similar ligand-loading procedure as for GQ.^21,22^ A saturated HQS solution was first prepared and sonicated for 2 hours for dispersion, then filtered (0.45 µm) to obtain a clean solution. Subsequently, 1.0 g of graphene oxide (GO) was added, and the mixture was stirred at room temperature for 48 hours to allow the sulfonated quinoline groups to anchor onto the GO surface. The resulting GQS composite was collected, thoroughly washed with deionized water until clear to remove excess HQS, and finally dried in an oven at 60 °C overnight.

### 2.3 Sorbent characterization

The fundamental structural, morphological, surface-chemical, and colloidal characteristics of the graphene oxide (GO) and its functionalized derivatives, GQ and GQS, were comprehensively evaluated through a suite of advanced analytical techniques. X-ray diffraction (XRD) was employed to ascertain the crystalline phases present in the materials. Measurements were conducted on a PANalytical X'Pert PRO diffractometer (Netherlands), utilizing Cu Kα radiation (*λ* = 1.5406 Å) across a 2*θ* range of 5–80°, with a scan step size of 0.02°. Functional groups and bonding interactions were identified using Fourier-transform infrared (FTIR) spectroscopy. Spectra were acquired on a PerkinElmer Spectrum One spectrometer (USA) within the wavenumber range of 4000–400 cm^−1^ at a resolution of 4 cm^−1^. The surface morphology and elemental composition were scrutinized *via* scanning electron microscopy (SEM) coupled with energy-dispersive X-ray spectroscopy (EDS). Prior to analysis, samples were sputter-coated with gold. Imaging and elemental mapping were performed on a JEOL JSM microscope operated at 20 kV. Textural properties, including specific surface area, pore volume, and pore size distribution, were determined from N_2_ adsorption–desorption isotherms measured at 77 K using a Quantachrome NOVA touch 4LX analyzer. The specific surface area was calculated according to the Brunauer–Emmett–Teller (BET) method, while pore-size distributions were derived from both the Barrett–Joyner–Halenda (BJH) and non-local density functional theory (NLDFT) models. Before analysis, all samples were degassed under vacuum at 120 °C for 6 h. The total pore volume was estimated at a relative pressure (*P*/*P*_0_) of 0.995. Finally, the colloidal stability and surface charge characteristics were assessed through dynamic light scattering (DLS) and zeta potential measurements. These analyses were performed using a Particle Sizing Systems instrument (Santa Barbara, USA). DLS measurements, which provide particle size distribution, were conducted with a 632.8 nm laser at 30 °C. Zeta potential, indicating the electrokinetic potential of the particles, was determined by electrophoretic light scattering at 25 °C with an applied electric field of 3.0 V cm^−1^.

### 2.4 Adsorption experiments

Batch adsorption experiments were conducted to evaluate the performance of GO, GQ, and GQS toward Cd(ii) under systematically varied operational conditions, including solution pH, sorbent dose, contact time, initial metal concentration, and temperature. All experiments were performed in polypropylene vessels using a thermostatically controlled orbital shaker (Scientific Precision Thermo-shaker, model SWB-27) operating at 150 rpm. Unless specified otherwise, all tests were carried out at a constant temperature of 25 ± 1 °C. pH-dependent studies were performed by adjusting the initial solution pH between 2 and 8 using diluted HCl or NaOH, while monitoring the final pH after equilibrium; pH values above 7 were avoided to prevent Cd(OH)_2_ precipitation.^27–29^ Sorbent-dose experiments were conducted by varying the adsorbent mass from 0.3 to 3.0 g L^−1^ at a fixed Cd(ii) concentration in order to determine the minimum dose required to achieve near-quantitative removal. After the predetermined contact time, the suspensions were filtered through 0.22 µm membrane filters, and the residual Cd(ii) concentrations (*C*_e_, mg L^−1^) were quantified using atomic absorption spectrophotometry (AAS; GBC 932 AA) at element-specific wavelengths. Instrument calibration was verified using procedural blanks and mid-range quality control standards to ensure analytical accuracy. All adsorption measurements were conducted under strictly identical conditions and in triplicate, and the mean values were reported when the relative standard deviation did not exceed 5%, confirming good experimental reproducibility.

Kinetic experiments were performed by contacting the sorbents with Cd(ii) solutions at the optimal pH and sorbent dose, followed by withdrawal of aliquots at predefined time intervals (5–600 min) to evaluate adsorption rate behavior. The resulting time-dependent data were analyzed using non-linear regression and fitted to the pseudo-first-order (PFO), pseudo-second-order (PSO), and Weber–Morris intraparticle diffusion models. Equilibrium adsorption isotherms were obtained using initial Cd(ii) concentrations ranging from 10 to 100 mg L^−1^ and interpreted using the Langmuir, Freundlich, and Sips models. Model performance was evaluated using the coefficient of determination (*R*^2^) and chi-square (*χ*^2^) statistics. The governing equations for kinetic, isotherm, and fitting analyses are summarized in Table S1.^41–45^ Temperature-dependent adsorption experiments were carried out over the range of 25–50 ± 1 °C, allowing thermodynamic parameters (Δ*G*°, Δ*H*°, and Δ*S*°) to be calculated using van't Hoff relationships, as detailed in Table S1.^46,47^

Desorption studies were conducted by treating Cd-loaded sorbents with 1.0 M HCl, HNO_3_, or H_2_SO_4_ under fresh batch conditions, after which the desorbed Cd(ii) concentration was determined to evaluate regeneration efficiency. The applicability of the sorbents to real wastewater was assessed using an industrial raffinate solution containing Cd(ii) and multiple co-existing ions. Optimized batch conditions (pH, dose, and contact time) were applied, and both target and competing ions were quantified before and after treatment to assess selectivity. Batch selectivity experiments are commonly used to compare preferential metal uptake under controlled conditions, whereas selectivity in continuous-flow systems may additionally depend on hydrodynamic and matrix-related effects. Accordingly, the real wastewater experiment is intended as a proof-of-concept validation of adsorption performance in a complex ionic matrix rather than as a comprehensive simulation of industrial-scale treatment. Performance metrics were calculated as removal efficiency *E*(%) = [(*C*_0_ − *C*_e_)/*C*_0_] × 100, equilibrium capacity *q*_e_ (mg g^−1^) = [(*C*_0_ − *C*_e_)*V*]/*m*, and distribution coefficient *K*_d_ (L g^−1^) = *q*_e_/*C*_e_, where *C*_0_ and *C*_e_ (mg L^−1^) are the initial and equilibrium concentrations, *V* (L) is the solution volume, and *m* (g) is the adsorbent mass.

## 3 Results & discussion

### 3.1 Sorbent characterization

#### 3.1.1 X-ray diffraction (XRD) analysis

**Fig. 1:**
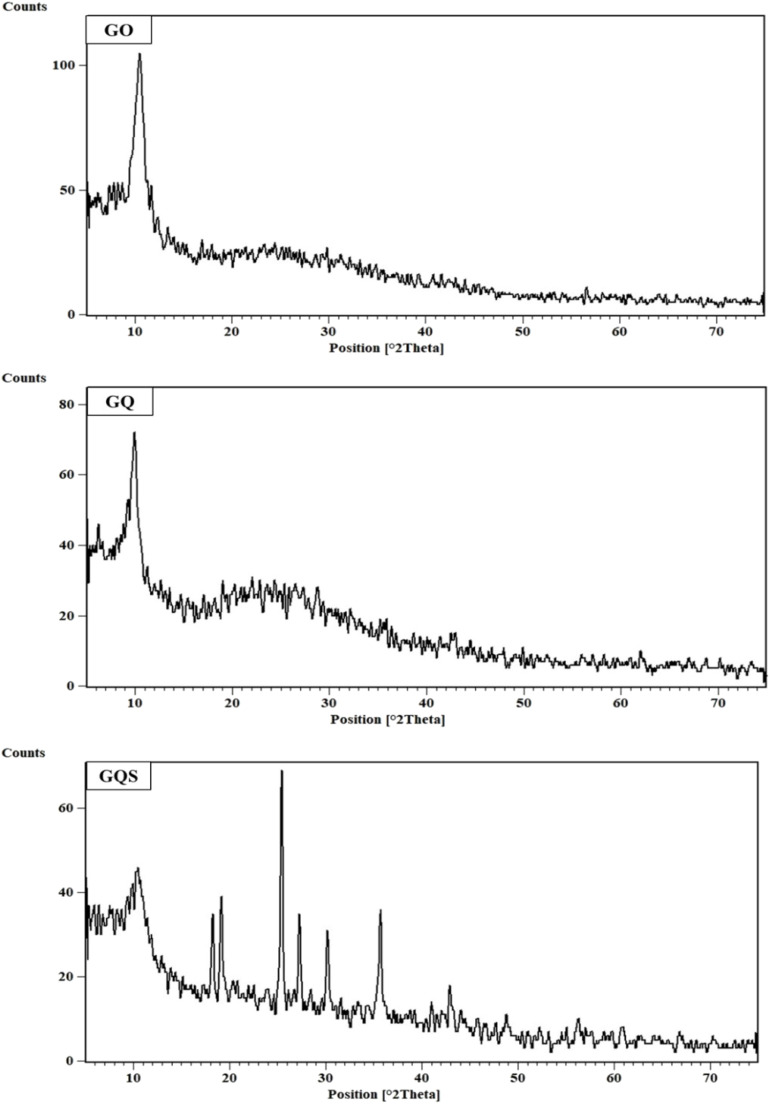
XRD patterns of graphene oxide (GO), GO functionalized with 8-hydroxyquinoline (GQ), and GO functionalized with 8-hydroxyquinoline-5-sulfonic acid (GQS) sorbents.

The structural integrity and crystallinity of the synthesized sorbents – graphene oxide (GO), GO functionalized with 8-hydroxyquinoline (GQ), and GO functionalized with 8-hydroxyquinoline-5-sulfonic acid (GQS) – were elucidated through X-ray Diffraction (XRD) analysis. As depicted in Fig. 1, the XRD pattern of GO exhibits a prominent and broad peak centered at approximately 10° 2*θ*. This characteristic peak is indicative of the layered structure of graphene oxide, corresponding to the (002) plane, and reflects the increased interlayer spacing attributed to the incorporation of oxygen-containing functional groups within its largely amorphous structure.^27–29^ The broadness of this peak also signifies the disordered nature of GO, a common feature arising from the various oxidation sites and structural defects.^27–29^ Such structural characteristics are advantageous for adsorption applications, as they contribute to a high surface area and abundant active sites for interaction with metal ions.^27–29^

Following functionalization, the GQ pattern similarly displays a broad peak around 10° 2*θ*, suggesting the retention of a graphitic structure after the attachment of 8-hydroxyquinoline.^20^ However, the GQ pattern appears broader and less intense compared to GO. This broader, less intense peak could be attributed to variations in the stacking order of graphene sheets or the presence of additional amorphous components introduced during the functionalization process.^20^ This structural modification likely results from the grafting of bulky quinoline groups onto the GO surface, which disrupts the regular stacking and introduces steric hindrance.^20^ Despite this increased disorder, the retention of a layered structure in GQ ensures the availability of active sites for Cd(ii) adsorption.

In stark contrast, the GQS sample, functionalized with 8-hydroxyquinoline-5-sulfonic acid, presents several distinct and sharper peaks. Notably, a strong peak at approximately 28° 2*θ* is observed, along with other crystalline phases between 20° and 40° 2*θ*.^48^ This significant alteration in the XRD pattern, with the emergence of well-defined peaks, strongly indicates a higher degree of crystallinity or molecular ordering in GQS compared to both GO and GQ. The presence of these sharp peaks suggests that functionalization with 8-hydroxyquinoline-5-sulfonic acid induces substantial structural modifications, potentially leading to better molecular packing or the formation of specific crystalline arrangements within the composite material.^48^ Sulfonated graphene-based materials are known to exhibit structural changes upon functionalization, sometimes leading to more defined XRD patterns, indicating successful incorporation and potentially enhanced properties.^48^ Collectively, these XRD patterns effectively highlight the structural evolution and modifications induced in GO upon functionalization, which are crucial for understanding their respective Cd(ii) adsorption capabilities.

#### 3.1.2 Fourier transform infrared (FTIR) analysis

**Fig. 2:**
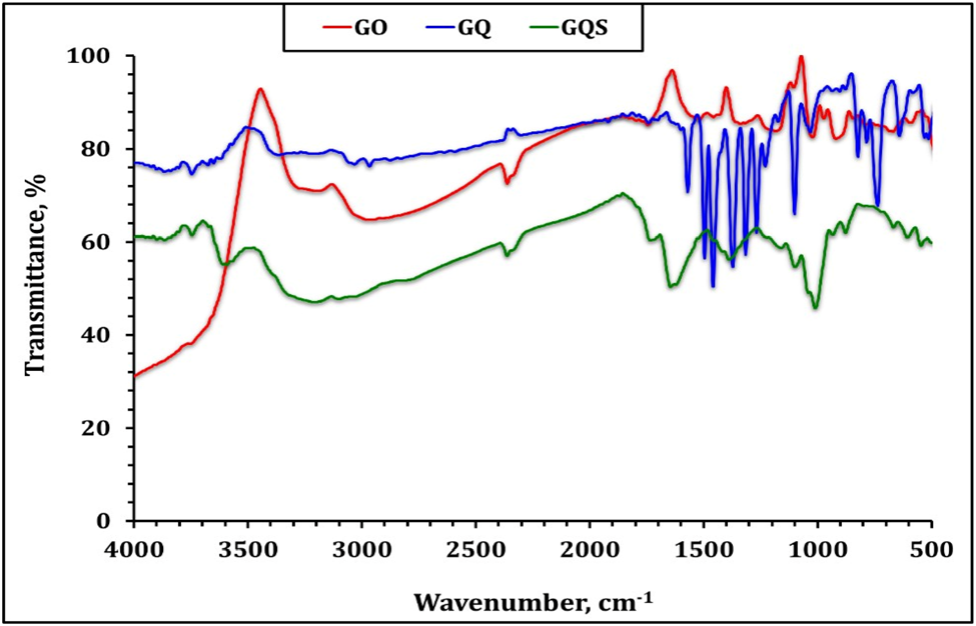
FTIR spectra of graphene oxide (GO), GO functionalized with 8-hydroxyquinoline (GQ), and GO functionalized with 8-hydroxyquinoline-5-sulfonic acid (GQS) sorbents.

The Fourier Transform Infrared (FTIR) spectra of graphene oxide (GO), GO functionalized with 8-hydroxyquinoline (GQ), and GO functionalized with 8-hydroxyquinoline-5-sulfonic acid (GQS) (Fig. 2) provide critical insights into the chemical modifications and functional groups present in the synthesized sorbents. The spectra clearly demonstrate the incorporation of nitrogen- and sulfur-containing functional groups and the progressive modification of the graphene oxide surface chemistry.^20,49,50^

For the GO spectrum, a broad absorption band observed around 3200–3500 cm^−1^ is attributed to the stretching vibrations of hydroxyl (–OH) groups, which are characteristic of the oxygen-containing functional groups on the GO surface.^27–29^ Additionally, the peak at approximately 1720 cm^−1^ corresponds to the stretching vibrations of carbonyl (C=O) groups, while the band near 1620 cm^−1^ is associated with the skeletal vibrations of the aromatic C=C bonds in the graphene structure.^27–29^ Peaks at 1220 cm^−1^ and 1050 cm^−1^ are indicative of C–O stretching vibrations, further confirming the presence of epoxy and alkoxy groups on the GO surface.^27–29^

After functionalization with 8-hydroxyquinoline, the FTIR spectrum of the GQ composite exhibits several notable changes that evidence successful grafting of the quinoline ligand. A distinct broadening and enhancement around ∼3130 cm^−1^ appears in GQ, which can be ascribed to aromatic =C–H stretching vibrations of the quinoline ring. This feature, together with a slight shift and decrease in the broad O–H band, indicates interaction between GO hydroxyl groups and the functional groups of 8-HQ.^20,21^ The marked reduction in intensity of the 3200–3500 cm^−1^ band suggests that a fraction of these hydroxyl groups participates in the functionalization process. New bands emerging at approximately 1570 cm^−1^ and 1470 cm^−1^ are assigned to C=N stretching and aromatic C=C stretching modes of the quinoline ring, respectively, consistent with the vibrational fingerprints of 8-HQ derivatives.^20,21^ In parallel, the diminished intensity of the C=O stretching band near ∼1710 cm^−1^ implies partial substitution, coordination, or hydrogen bonding involving GO carboxyl groups during ligand attachment. Furthermore, the band observed around 1280 cm^−1^ is associated with C–N and aromatic C–O vibrations originating from the phenolic functionality of 8-HQ, providing additional evidence for the immobilization of quinoline species on the GO framework.^20,21^

In the case of GQS, functionalization with 8-hydroxyquinoline-5-sulfonic acid induces further modifications to the GO spectrum that reflect the introduction of sulfonated quinoline moieties. As in GQ, the appearance of bands in the 1570–1470 cm^−1^ region confirms the presence of the quinoline ring through C=N and aromatic C=C stretching modes. However, GQS exhibits additional, more intense bands in the 1200–1040 cm^−1^ range, which are characteristic of S=O and O–S–O stretching vibrations of sulfonic groups (–SO_3_H/–SO_3_^−^).^48–50^ These sulfonate-related bands, together with a broadened O–H stretching envelope, indicate the incorporation of strongly acidic sulfonic functionalities that enhance hydrogen-bonding and electrostatic interaction capability.^48–50^ As with GQ, a decrease in the intensity of the GO C=O band and subtle changes in the C–O region suggest that both carboxyl and hydroxyl groups are involved in anchoring the HQS ligand. Overall, the combined spectral features of GQ and GQS—namely the emergence of quinoline-specific C=N/C=C bands, the new aromatic C–O/C–N vibrations, and, in the case of GQS, pronounced S=O/O–S–O signals—confirm the successful functionalization of GO with 8-HQ and 8-HQ-5-sulfonic acid and substantiate the formation of quinoline-rich, ligand-bearing sorbent surfaces. The presence of these functional groups is expected to play a pivotal role in the adsorption mechanism, which will be further discussed in subsequent sections.

#### 3.1.3 Scanning electron microscopy-energy dispersive X-ray spectroscopy analysis

**Fig. 3:**
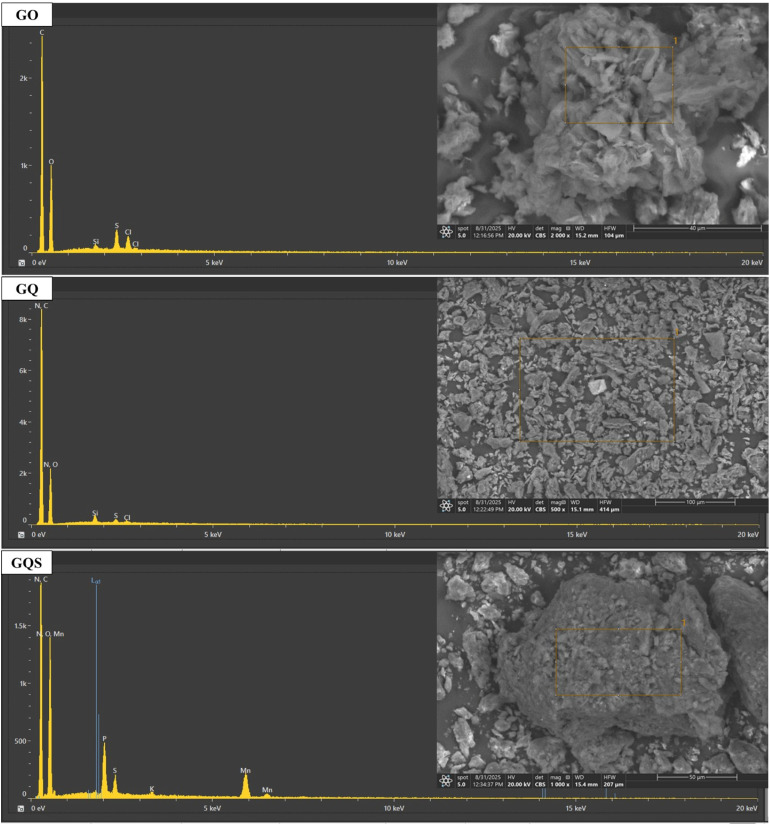
SEM-EDS spectrum of graphene oxide (GO), GO functionalized with 8-hydroxyquinoline (GQ), and GO functionalized with 8-hydroxyquinoline-5-sulfonic acid (GQS) sorbents.

SEM-EDS analysis provides essential insights into the morphological and elemental characteristics of graphene oxide (GO), 8-hydroxyquinoline functionalized GO (GQ), and 8-hydroxyquinoline-5-sulfonic acid functionalized GO (GQS) (Fig. 3). These analyses are crucial for understanding structural and chemical modifications and their potential impact on Cd(ii) adsorption.

The SEM images of GO reveal a morphology dominated by irregularly shaped, aggregated particles. This aggregation is characteristic of graphene oxide due to strong van der Waals forces and hydrogen bonding between the layers, often leading to a stacked morphology.^30–32^ While aggregated, this morphology typically still presents a high surface area and facilitates the accessibility of oxygenated functional groups, which are critical for adsorption applications.^30–32^ The EDS spectrum of GO confirms the predominant presence of carbon (C) and oxygen (O), attributed to the sp^2^-hybridized carbon framework and various oxygen-containing functional groups (*e.g.*, hydroxyl, epoxy, and carboxyl groups).^30–32^ Minor peaks for silicon (Si), sulfur (S), and chlorine (Cl) are also observed, likely originating from impurities or residual reagents used during synthesis. The high oxygen content in GO highlights its hydrophilic nature and the abundance of active sites available for Cd(ii) adsorption through electrostatic attraction and complexation mechanisms.

In contrast, the SEM images of the GQ composite show a more uniform distribution of smaller, plate-like particles compared to the larger agglomerates in the GO, indicating that 8-hydroxyquinoline functionalization alters the surface morphology and reduces sheet aggregation.^20–22^ The presence of additional grooves, folds, and surface voids suggests an increased microscale surface area, which is favorable for Cd^2+^ adsorption.^20–22^ The EDS spectrum of GQ shows prominent peaks for carbon (C) and nitrogen (N), along with oxygen (O). The significant presence of nitrogen confirms the successful incorporation of 8-hydroxyquinoline onto the GO surface.^20–22^ Nitrogen-containing functional groups, such as quinoline moieties, are expected to enhance the sorbent's affinity for Cd(ii) ions through chelation and possibly π–π interactions.^20–22^ The reduced oxygen content in GQ compared to GO suggests that some oxygen-containing groups were either consumed or masked during functionalization.

The SEM images of GQS reveal a morphology characterized by the formation of distinct particle agglomerates, differing from the structures observed in both GO and GQ. These agglomerates may result from the strong intermolecular interactions induced by the introduction of sulfonic acid groups, which can promote particle clustering.^49,50^ The EDS spectrum of GQS confirms the presence of carbon (C), nitrogen (N), and oxygen (O), along with a significant additional peak for sulfur (S). The sulfur peak is directly attributed to the sulfonic acid groups introduced during functionalization with 8-hydroxyquinoline-5-sulfonic acid, unequivocally confirming successful modification.^49,50^ These groups significantly enhance the sorbent's acidity and provide additional active sites for Cd(ii) adsorption through ion exchange and coordination mechanisms.^49,50^ The detection of minor manganese (Mn), phosphorus (P), and potassium (K) may be due to residual reagents or impurities from the synthesis and functionalization processes.

The comparative analysis of the SEM-EDS results highlights the crucial structural and compositional differences among the three sorbents. GO exhibits a relatively simple structure with abundant oxygen-containing functional groups, which primarily facilitate Cd(ii) adsorption through electrostatic interactions. Functionalization with 8-hydroxyquinoline in GQ introduces nitrogen-containing groups that enhance the sorbent's chelation ability and potentially reduce particle aggregation, thereby increasing the available active sites. Further functionalization with 8-hydroxyquinoline-5-sulfonic acid in GQS introduces highly acidic sulfonic acid groups, which not only increase the sorbent's acidity but also provide additional strong binding sites for Cd(ii) adsorption.

#### 3.1.4 Textural and structural characterization of sorbents

The textural and structural properties of the synthesized sorbents—graphene oxide (GO), GO functionalized with 8-hydroxyquinoline (GQ), and GO functionalized with 8-hydroxyquinoline-5-sulfonic acid (GQS)—were comprehensively evaluated using Brunauer–Emmett–Teller (BET) and Density Functional Theory (DFT) analyses (Fig. S1). These techniques are crucial for providing insights into surface area, pore size distribution, and pore volume, all of which are key parameters influencing the adsorption performance of materials.

The multi-point BET analysis (Fig. S1 and Table 1) revealed that graphene oxide (GO) exhibits the highest specific surface area, recorded at 75.30 m^2^ g^−1^. This indicates strong adsorbate–adsorbent interactions on a relatively homogeneous surface, characteristic of well-exfoliated GO.^25,26^ In contrast, functionalization markedly altered these properties: GO functionalized with 8-hydroxyquinoline (GQ) displayed a reduced surface area of 34.74 m^2^ g^−1^, and GO functionalized with 8-hydroxyquinoline-5-sulfonic acid (GQS) showed an even lower value of 23.47 m^2^ g^−1^. This trend of reduced surface area reflects partial pore blocking and an increase in surface heterogeneity caused by the grafting of bulky organic moieties, a phenomenon consistent with previous reports on organic-modified graphene derivatives.^20,21,25,26^ Although functionalization decreases the BET surface area, the concurrent introduction of chemically specific N,O- and N,O/S-donor chelating sites outweighs these textural losses, demonstrating that Cd(ii) uptake in GQ and GQS is governed primarily by coordination chemistry rather than surface area alone.

**Table 1:** Surface parameters of graphene oxide (GO), GO functionalized with 8-hydroxyquinoline (GQ), and GO functionalized with 8-hydroxyquinoline-5-sulfonic acid (GQS) sorbents

Sample	Total pore volume, cm^3^ g^−1^	Average pore size, nm	Surface area (m^2^ g^−1^)
GO	0.0909	1.925	75.3
GQ	0.0503	1.921	34.7
GQS	0.0452	1.928	23.4

Barrett–Joyner–Halenda (BJH) pore-size distribution analysis (Table 1) confirms that all three materials exhibit predominantly mesoporous structures, with pore widths falling within the 2–50 nm range defined by IUPAC classification.^20,25^ Consistent with this, the nitrogen adsorption–desorption isotherms for GO, GQ, and GQS display type V behavior with H1 hysteresis loops in the *P*/*P*_0_ range of 0.5–0.9,^21,26^ indicative of slit-shaped mesopores and relatively uniform pore channels. The GO shows a total pore volume of 0.0909 cm^3^ g^−1^ and a mode pore width of 1.89 nm, placing it near the micro–mesopore transition region, with a broad distribution extending up to ∼30 nm. Following functionalization, both GQ and GQS exhibit reduced total pore volumes (0.0503 and 0.0452 cm^3^ g^−1^, respectively), accompanied by an increase in the mode pore width to 2.94 nm. This shift toward larger pore sizes suggests that the incorporation of 8-hydroxyquinoline and sulfonic acid groups disrupts some of the narrower pores, partially widening existing mesoporous channels.^20,21,25,26^

Collectively, the BET and BJH data underscore a significant trade-off: while GO offers the greatest surface area and pore volume, which are generally favorable for high adsorption capacity, it primarily relies on oxygen-containing functional groups and electrostatic interactions. Conversely, the functionalized materials, GQ and GQS, despite their reduced textural metrics, furnish specific nitrogen- and oxygen-donor ligands (from 8-hydroxyquinoline) and highly acidic sulfonic acid groups. These functional groups are anticipated to enable stronger and more selective coordination with Cd(ii) ions. Importantly, the retained mesoporosity in both GQ and GQS (with a mode pore width of 2.94 nm) ensures facile mass transport of Cd(ii) ions to the interior binding sites, thereby mitigating diffusional limitations that commonly affect microporous adsorbents.^20,21^ This strategic functionalization, despite surface area reduction, therefore enhances targeted Cd(ii) adsorption by favoring ligand-controlled inner-sphere complexation over purely textural effects.

#### 3.1.5 Particle size and zeta potential analysis

Dynamic light scattering (DLS) and zeta potential measurements were performed to evaluate the hydrodynamic particle size, and surface charge of the three sorbents—GO, GQ, and GQS. These parameters provide essential insight into colloidal stability, aggregation behavior, and surface chemistry, all of which strongly influence adsorption performance in aqueous media.

DLS results (Fig. S2 and Table 2) reveal distinct differences among the three sorbents. GO exhibits a mean hydrodynamic diameter of 1285.9 nm, reflecting moderately stable, sheet-like structures that remain partially aggregated in water.^23^ Upon functionalization with 8-hydroxyquinoline, the mean particle size increases markedly to 1512.8 nm, indicating enhanced aggregation due to π–π stacking, hydrogen bonding, or reduced electrostatic repulsion introduced by the neutral quinoline ligand. In contrast, the GQS sorbent shows a substantially smaller particle size of 379.0 nm, reflecting a more stable and better-dispersed colloidal system. This improved dispersion is attributed to the presence of deprotonated sulfonic groups (–SO_3_^−^) that enhance electrostatic repulsion, thus reducing aggregation and yielding a more monodisperse suspension. These trends demonstrate that functionalization affects not only the chemistry but also the dispersion and aggregation behavior of graphene-based sorbents.

**Table 2:** Surface parameters of graphene oxide (GO), GO functionalized with 8-hydroxyquinoline (GQ), and GO functionalized with 8-hydroxyquinoline-5-sulfonic acid (GQS) sorbents

Sample	DLS analysis, nm	Zeta potential, mV
GO	1285.9	−17.04
GQ	1512.8	−15.11
GQS	379.0	−21.14

The zeta potential values further support these observations. GO exhibits a moderately negative surface charge (−17.04 mV) due to its abundant –COOH and –OH groups.^23^ Functionalization with 8-hydroxyquinoline reduces the magnitude of the negative charge to −15.11 mV, consistent with partial masking or replacement of oxygenated moieties by neutral quinoline groups. Conversely, GQS shows a significantly more negative zeta potential (−21.14 mV), confirming the incorporation of strongly acidic sulfonic groups that increase surface charge density. All three surfaces fall within the range of moderate colloidal stability; however, the more negative zeta potential of GQS explains its enhanced dispersion, whereas the reduced charge in GQ correlates with its larger hydrodynamic aggregates. Overall, the combined DLS and zeta potential data reveal that ligand functionalization profoundly influences colloidal behavior. While GQ forms larger aggregates due to reduced surface charge and enhanced aromatic interactions, GQS maintains high dispersion stability due to its sulfonate-rich surface. These physicochemical characteristics directly affect the accessibility of active sites and help explain differences in adsorption performance among the three sorbents.

In summary, the comprehensive characterization of GO, GQ, and GQS using XRD, FTIR, SEM-EDS, BET, DFT, DLS, and zeta potential analyses confirms the successful functionalization of graphene oxide and clarifies the structural, chemical, textural, and surface-charge modifications induced by ligand incorporation. XRD analysis demonstrated a clear evolution from the disordered, layered architecture of GO to a more organized structure in GQ and particularly in GQS, reflecting the structural rearrangements accompanying quinoline and sulfonated quinoline grafting. FTIR spectra provided definitive evidence for the introduction of 8-hydroxyquinoline and 8-hydroxyquinoline-5-sulfonic acid, as indicated by the appearance of characteristic C=N, aromatic C=C, and S=O stretching vibrations. SEM-EDS results further supported these findings by revealing distinct morphological transformations and confirming the presence of nitrogen and sulfur, unequivocally validating the successful attachment of the respective ligands onto the GO framework. Although BET and DFT analyses indicated reductions in specific surface area and pore volume following functionalization, they also confirmed the preservation of mesoporosity—an essential feature for efficient mass transport and adsorptive accessibility. Complementary DLS and zeta potential measurements revealed changes in particle size and surface charge, with functionalization increasing hydrodynamic diameter in GQ and enhancing colloidal stability in GQS through more negative zeta potential values. Collectively, these integrated results demonstrate that, despite moderate alterations to physical surface characteristics, functionalization effectively introduces nitrogen- and sulfur-rich chelating moieties that substantially enhance the affinity of the modified materials toward Cd(ii). This strategic tuning of chemical functionality therefore represents a key factor governing the superior adsorption performance of the engineered graphene oxide sorbents.

### 3.2 Process of Cd(ii) adsorption

#### 3.2.1 Impact of solution pH

**Fig. 4:**
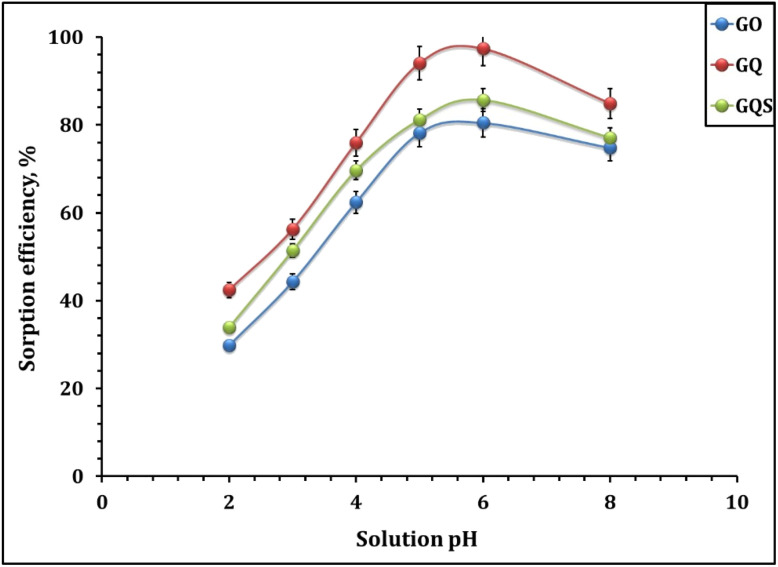
Impact of solution pH on Cd(ii) sorption efficiency: sorbent dose 2.0 g L^−1^, time 240 min, initial concentration of 50 mg L^−1^, 25 °C.

The effect of solution pH on Cd(ii) adsorption by GO, GQ, and GQS was evaluated over the pH range 2–8 under fixed experimental conditions of 2.0 g L^−1^ sorbent dose, 50 mg L^−1^ initial Cd(ii) concentration, 240 min shaking time, and room temperature (Fig. 4). The adsorption efficiency of all three sorbents exhibits a pronounced dependence on pH, reflecting the coupled influence of Cd(ii) aqueous speciation, surface protonation–deprotonation equilibria, and the chemical functionalities introduced by GO modification. Cadmium speciation analysis as a function of pH (Fig. S3) indicates that Cd(ii) exists predominantly as free Cd^2+^ over the investigated pH range, with hydrolysis becoming increasingly relevant toward the alkaline end.^51,52^ Within the working pH window of 2–7, no significant formation of Cd(OH)_2_(s) is expected; therefore, the observed Cd removal can be attributed primarily to adsorption and chelation processes rather than precipitation. In parallel, zeta potential measurements (Table 2) show that GO, GQ, and GQS possess negatively charged surfaces, suggesting a strong inherent tendency toward electrostatic attraction with Cd(ii) species, particularly once surface deprotonation begins to dominate.^21–24^ From an application perspective, this pH window (=4–6) is highly relevant to industrial and municipal wastewaters, which are often near-neutral or only mildly acidic; therefore, high performance at pH = 6 reduces the extent of chemical pH adjustment required in practical treatment trains.

At very low pH (2–3), adsorption capacities remain low for all sorbents due to extensive protonation of surface oxygenated groups (carboxyl and hydroxyl) on GO and the quinoline nitrogen and sulfonic acid functionalities present in GQ and GQS. Protonation diminishes the availability of electron-donating groups for coordination and imparts a neutral or even positively charged surface character, thereby generating electrostatic repulsion toward Cd^2+^ while also intensifying competition between H^+^ and Cd(ii) for active sites.^21–24^ This interpretation aligns with FTIR results, which confirm the abundance of protonatable groups whose coordination capacity is highly sensitive to pH-dependent dissociation equilibria. As the pH increases to 3–6, progressive deprotonation of these functional groups enhances the density of negatively charged sites, thereby increasing both electrostatic attraction and the likelihood of inner-sphere complexation with Cd(ii).^51,52^ In this intermediate pH region, oxygenated groups on GO become more nucleophilic, while the N-donor centers of GQ and the strongly acidic sulfonated sites of GQS increasingly contribute to metal binding.^21–24^ At pH 6, all materials exhibit their maximum adsorption capacity, corresponding to (i) the predominance of free Cd^2+^ in solution, and (ii) the effective deprotonation of carboxylate, phenolate, quinoline-N, and sulfonate groups, which together generate an optimal landscape of electrostatic and coordinative interactions.^21–24^ Notably, achieving maximum uptake at pH = 6 is advantageous for wastewater treatment because it is close to the operational pH of many effluents and avoids the need for strongly alkaline conditions that may induce metal hydroxide precipitation and complicate sludge handling. Beyond pH 6.0, the slight decrease in adsorption capacity is more consistently attributed to the onset of Cd(ii) hydrolysis and the increasing contribution of soluble hydroxo species (*e.g.*, Cd(OH)^+^), which may exhibit different coordination preferences and lower effective affinity toward the surface binding sites compared with free Cd^2+^.^51,52^ In addition, the emergence of hydrolyzed species can modify interfacial speciation and competition at the solid–solution boundary, leading to a reduced fraction of strongly adsorbed Cd under otherwise identical conditions. Graphene oxide (GO),^23^ amine-functionalized GO materials,^24^ a GO/paper hybrid prepared from waste fibers,^25^ and cyclodextrin-GO hybrids^31^ all exhibited comparable sorption efficacy (pH-dependent) during Cd(ii) adsorption process. Accordingly, the pH-dependent trend observed here provides direct operational guidance for real effluents: treatment at mildly acidic-to-near-neutral pH maximizes Cd(ii) capture while maintaining adsorption-dominated removal rather than precipitation-driven artifacts.

#### 3.2.2 Impact of sorbent dosage

**Fig. 5:**
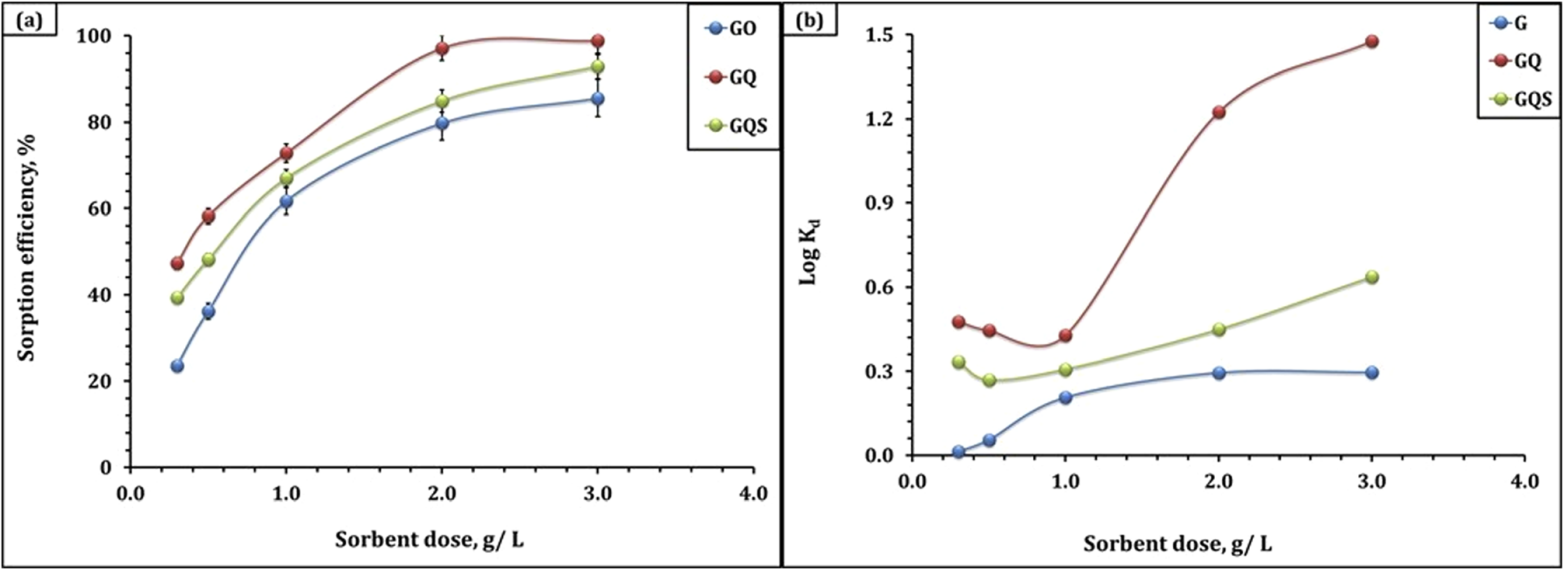
Impact of various conditions on Cd(ii) sorption efficiency (a) dose: solution pH of 4.98, time 240 min, initial concentration of 50 mg L^−1^, 25 °C; (b) distribution coefficient (log *K*_d_) on sorbents dose (pH 5.9; time of 240 min; original concentration: 50 mg L^−1^; and room temperature).

The effect of sorbent dose on Cd(ii) uptake by GO, GQ, and GQS was investigated in the range 0.3–3.0 g L^−1^ at fixed initial Cd(ii) concentration (50 mg L^−1^), contact time (240 min), and room temperature, under the previously optimized pH conditions (Fig. 5 and Table S2). The experimental data show that, for all three sorbents, the removal efficiency (% *R*) increases markedly with increasing sorbent dose, while the equilibrium adsorption capacity (*q*_e_, mg g^−1^) exhibits the opposite trend, decreasing as the dose increases. At the lowest dose of 0.3 g L^−1^, Cd(ii) removal is relatively modest, with % *R* values of 23.6, 47.3, and 39.3% for GO, GQ, and GQS, respectively, but the corresponding capacities are high, reaching 39.3, 78.8, and 65.5 mg g^−1^. This behavior is typical of batch adsorption systems, where a small mass of sorbent is exposed to a relatively large number of Cd(ii) ions, leading to a high degree of site utilization and a large amount of metal adsorbed per unit mass.^23–26^ As the dose is increased from 0.3 to 3.0 g L^−1^, the removal efficiencies increase steadily to 85.6% (GO), 98.9% (GQ), and 92.9% (GQS), reflecting a progressively larger total number of available binding sites that can capture a greater fraction of Cd(ii) from solution. In contrast, *q*_e_ decreases systematically with increasing dose, dropping to 14.3, 16.5, and 15.5 mg g^−1^ for GO, GQ, and GQS at 3.0 g L^−1^, due to the reduced ratio of Cd(ii) ions to available surface sites and the fact that many active sites remain unsaturated at higher sorbent dosages.^23–26^ These trends underscore the critical need for optimizing the sorbent dose to achieve an ideal balance between maximizing removal efficiency and maintaining practical capacity and economic viability for a given application. Based on this dose-optimization analysis, a sorbent dose of 2.0 g L^−1^ was selected for subsequent batch experiments, as it provides near-maximum Cd(ii) removal (=80–97% for GO, GQ, and GQS) while maintaining reasonable adsorption capacities (=20–24 mg g^−1^) and experimental practicality.

The variation of the distribution coefficient (*K*_d_) with sorbent dose offers additional insight into the affinity of each sorbent for Cd(ii), and its magnitude is directly related to the surface complexation ability of the material (Fig. 5).^51,52^ Sorbents containing stronger or more abundant coordinating functionalities (*e.g.*, quinoline-N and sulfonate groups in GQ and GQS) produce lower equilibrium concentrations (*C*_e_) and therefore higher *K*_d_ values. Accordingly, the markedly higher *K*_d_ values observed for GQ reflect its enhanced inner-sphere complexation capacity arising from its dense population of N- and O-donor chelating sites, whereas GQS shows intermediate affinity and GO exhibits the lowest *K*_d_ due to the reliance on weaker oxygenated groups.

#### 3.2.3 Reaction time and kinetic studies

**Fig. 6:**
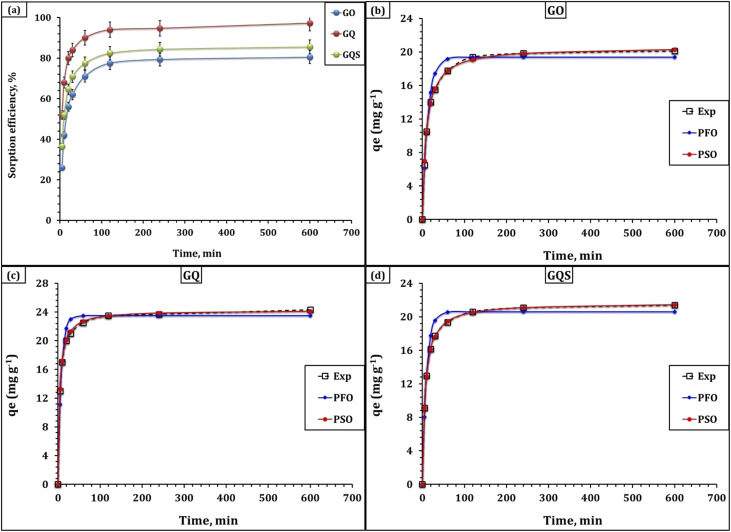
(a) Effect of contact time on the sorption efficiency of Cd(ii) ions; (b) PFO and PSO kinetic fit for GO sorbent; (c) PFO and PSO kinetic fit for GQ sorbent; (d) PFO and PSO kinetic fit for GQS sorbent. Experimental conditions: initial ion concentration = 50 mg L^−1^, pH = 6.01, dose = 2.0 g L^−1^, temperature = 25 °C.

The effect of contact time on Cd(ii) adsorption by GO, GQ, and GQS was investigated over the range 5–600 min at fixed initial Cd(ii) concentration (50 mg L^−1^), sorbent dose (2.0 g L^−1^), optimized pH, and room temperature (Fig. 6a). The time-dependent uptake profiles for GO, GQ and GQS reveal a similar general trend for all three sorbents: a very rapid initial adsorption stage followed by a slower approach to equilibrium. Within the first 5 min, Cd(ii) uptake already reaches 26.0, 52.0, and 36.5% for GO, GQ, and GQS, respectively, corresponding to capacities of 6.5, 13.0, and 9.1 mg g^−1^, indicating that a large fraction of the total driving force is consumed in the early stages of contact. This fast initial uptake is attributed to the abundant availability of vacant, energetically favorable binding sites at the external surface and near-surface regions of the sorbents.^23,24^ As contact time extends from 30 to 120 min, the removal efficiencies grow steadily and more gradually, reaching 62.0–77.5% (GO) and 84.0–94.0% (GQ) and 70.9–82.4% (GQS), respectively, which corresponds to capacities in the range 15.5–19.4 mg g^−1^ for GO, 21.0–23.5 mg g^−1^ for GQ, and 17.7–20.6 mg g^−1^ for GQS. Beyond 120–240 min, the uptake curves progressively level off, with only marginal increases observed up to 600 min, indicating that near-equilibrium is approached by 240 min for all sorbents (*q*_*t*_ = 19.8, 23.7, and 21.1 mg g^−1^ for GO, GQ, and GQS, respectively). From a practical standpoint, the time required to reach approximately 90% of the equilibrium uptake (*t*_90_) is about 60 min for GQ, 120 min for GQS, and 180–240 min for GO, highlighting the rapid adsorption kinetics of the quinoline-functionalized sorbents relative to pristine GO. The gradual deceleration in adsorption rate with time is typical of sorption processes in which (i) the most reactive sites become progressively occupied, (ii) diffusion pathways lengthen as Cd(ii) migrates into deeper pores or interior regions, and (iii) electrostatic repulsion between adsorbed Cd(ii) and Cd^2+^ in solution increases.^23–27^ The faster approach to equilibrium and higher capacities observed for GQ and GQS compared with GO are consistent with their richer population of N- and S-containing functional groups and more favorable electrostatic environments, as evidenced by FTIR, SEM-EDS, zeta potential, and BET analyses.

The kinetic data were further analyzed using pseudo-first-order (PFO), pseudo-second-order (PSO), and Weber–Morris intraparticle diffusion models (Fig. 6b–d) and displayed in Table 3.^41–43^ For the PFO model, the calculated equilibrium capacities (*q*_1_) are 19.4, 23.5, and 20.6 mg g^−1^ for GO, GQ, and GQS, respectively, with rate constants *k*_1_ of 0.076, 0.129, and 0.099 min^−1^. Although the PFO model yields reasonably good correlation coefficients (*R*^2^ = 0.96, 0.93, and 0.94), the higher *χ*^2^ values (0.47–0.69) and the slight mismatch between *q*_1_ and the experimental *q*_e_ indicate that it does not best describe the system. In contrast, the PSO model provides an excellent fit for all three sorbents, with *R*^2^ = 0.99 and very low *χ*^2^ values (0.02–0.05), and the calculated *q*_2_ values (20.6, 24.3, and 21.7 mg g^−1^) are in close agreement with the experimental equilibrium capacities. Taken together, the superior PSO fit (higher *R*^2^, lower *χ*^2^, and closer agreement between *q*_2_ and *q*_e_) indicates that the uptake kinetics are strongly influenced by surface-reaction-controlled processes involving specific binding sites; however, this behavior should be interpreted as being consistent with a chemisorption contribution to the overall adsorption mechanism.^41–43^ In practical terms, the PSO model provides a useful kinetic description of the dependence of uptake rate on the density and accessibility of reactive surface sites (*e.g.*, –COO^−^, –OH, quinoline N/O, and –SO_3_^−^), whereas the PFO model is more appropriate when adsorption is dominated by weak, non-specific physical interactions; this explains the closer agreement between *q*_2_ and *q*_e_ and the lower *χ*^2^ values obtained for PSO. It is worth noted that, the PSO rate constants *k*_2_ follow the order GQ (0.010) > GQS (0.007) > GO (0.005 min^−1^), and the initial adsorption rate (*h*) is also highest for GQ (5.9) compared to GQS (3.2) and GO (2.1). Moreover, the PSO half-times (*t*_1/2_) of 4.1 h for GQ and 6.9 and 9.7 h for GQS and GO, respectively, clearly indicate that GQ not only attains higher equilibrium uptake but does so in a shorter time frame, which is in consistent with the insights from the experimental results.

**Table 3:** The values of the parameters used in the applied kinetics model

	GO	GQ	GQS
**Pseudo first-order model**			
*q*_1_ (mg g^−1^)	19.4	23.5	20.6
*k*_1_ (min^−1^)	0.076	0.129	0.099
*R* ^2^	0.96	0.93	0.94
*X* ^2^	0.47	0.69	0.57

**Pseudo second-order model**			
*q*_2_ (mg g^−1^)	20.6	24.3	21.7
*k*_2_ (min^−1^)	0.005	0.010	0.007
*h* (mg g^−1^ min^−1^)	2.1	5.9	3.2
*t*_1/2_ (min)	9.7	4.1	6.9
*R* ^2^	0.99	0.99	0.99
*X* ^2^	0.05	0.02	0.02

The Weber–Morris intraparticle diffusion model provides additional insight into the diffusion-controlled aspects of the adsorption process (Fig. S4 and Table S3).^41–43^ The intraparticle diffusion rate constants (*k*_i_) are 1.18, 2.04, and 0.45 mg g^−1^ min^−1/2^ for GO, GQ, and GQS, respectively, with corresponding intercepts (*C*) of 24.7, 64.3, and 14.1 and high correlation coefficients (*R*^2^ = 0.98–0.99). The non-zero intercepts indicate that intraparticle diffusion is not the sole rate-limiting step but operates in conjunction with a significant boundary-layer (film diffusion) contribution.^23–27^ The comparatively larger *k*_i_ for GQ suggests that, once Cd(ii) reaches the particle surface, its diffusion into the interior and adsorption onto internal sites proceeds more rapidly than for GO and GQS, which is consistent with the higher density of strong chelating sites identified by FTIR and EDS. The relatively high *C* values, especially for GQ, further reflect the contribution of a fast initial surface-controlled stage before intraparticle diffusion becomes predominant.^23–27^ These kinetic insights, when considered alongside the structural and surface-chemical characterization, support a multi-mechanistic adsorption process in which surface-reaction-controlled uptake plays an important role, while intraparticle diffusion and boundary-layer effects contribute to the overall rate behavior. Accordingly, the overall uptake kinetics reflect a coupled mechanism involving rapid boundary-layer/surface interaction followed by slower diffusion into mesoporous domains.

Overall, the kinetic evaluation indicates that Cd(ii) adsorption on graphene oxide and quinoline-functionalized graphene oxide sorbents follows a multi-stage process dominated by chemisorption, with intraparticle diffusion acting as a secondary rate-influencing step. Across the literature, sorbents belonging to markedly different material classes show a striking convergence in their kinetic signatures for Cd(ii) adsorption. Despite their structural diversity, these systems consistently exhibit rate profiles dominated by chemisorption-controlled steps, and diffusion through internal pores or particulate channels represents an integral secondary stage, modulating the rate but not determining the overall mechanism. This dual contribution–surface reaction as the governing step and intra-particle diffusion as a supporting one—has emerged as a recurrent kinetic pattern across numerous Cd(ii) sorbent systems. Sorbents demonstrating this behavior include oxide–polyamide hybrids such as graphene oxide (GO),^23^ amine-functionalized GO materials,^24^ A GO/paper hybrid prepared from waste fibers,^25^ Magnetic GO composites—including GO-tryptophan (MGO-Trp),^26^ α-Fe_2_O_3_/GO,^27^ few-layered magnetic graphene oxide (FLMGO),^28^ and PAN = Fe_3_O_4_@GO,^29^ and cyclodextrin-GO hybrids.^31^

#### 3.2.4 Impact of initial concentration and isotherm investigation

**Fig. 7:**
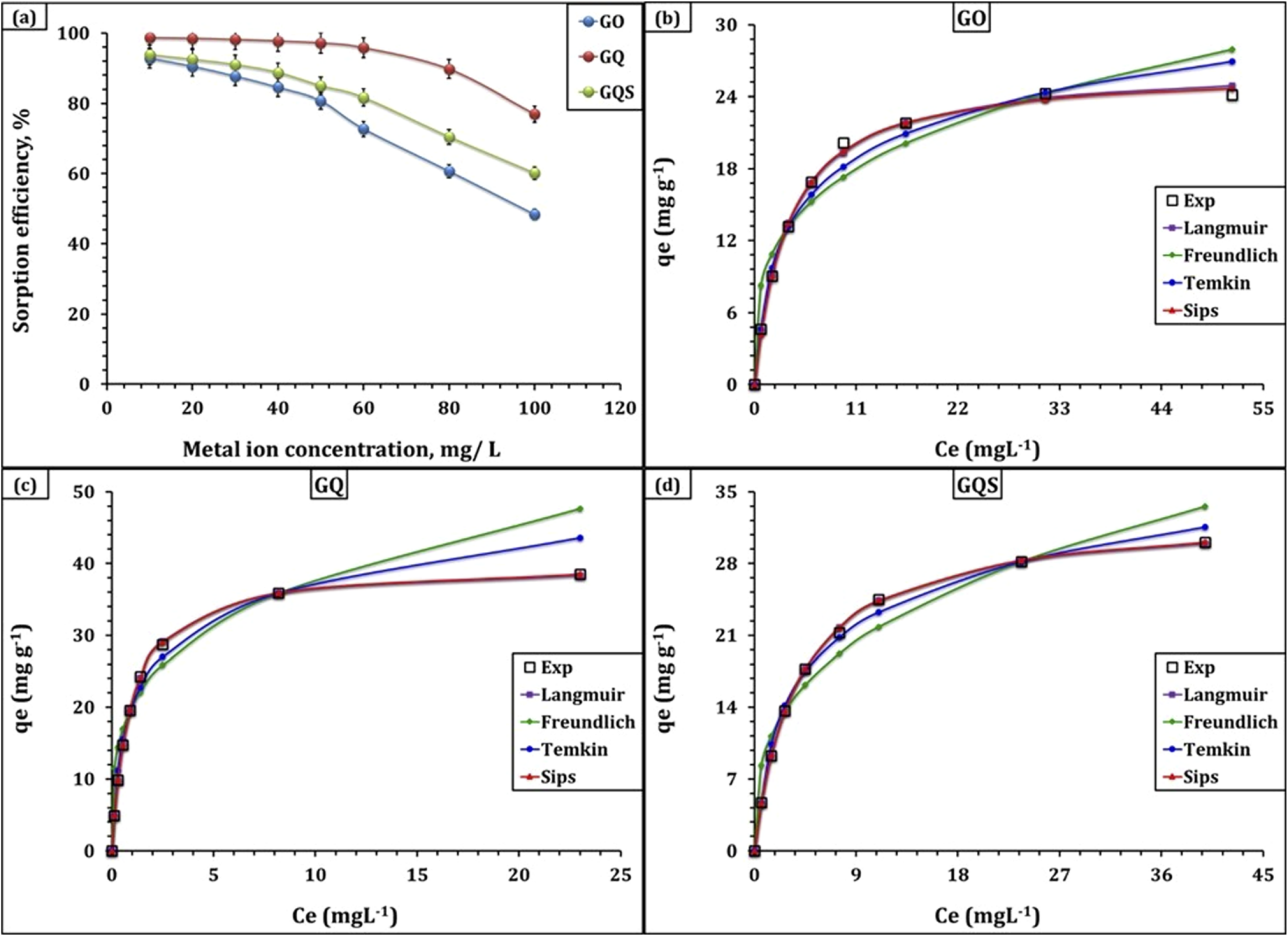
(a) Effect of initial concentration on the sorption efficiency of Cd(ii) ions; (b) isotherm profile for GO sorbent; (c) isotherm profile for GQ sorbent; (d) isotherm profile for GQS sorbent. Experimental conditions: time = 240 min, pH = 6.00, dose = 2.0 g L^−1^, temperature = 25 °C.

The effect of initial Cd(ii) concentration on the adsorption performance of GO, GQ, and GQS was investigated over the range 10–100 mg L^−1^ at fixed sorbent dose, contact time, and temperature (Fig. 7a). The experimental data show that, for all three sorbents, the removal efficiency (% *R*) decreases with increasing initial concentration, whereas the equilibrium adsorption capacity (*q*_e_, mg g^−1^) increases. At low concentration (10 mg L^−1^), very high removal efficiencies of 92.8, 98.7, and 93.8% are achieved on GO, GQ, and GQS, respectively, with relatively modest *q*_e_ values of 4.6, 4.9, and 4.7 mg g^−1^. As the initial Cd(ii) concentration increases to 50 mg L^−1^, % *R* decreases to 80.7, 97.2, and 85.0%, while qe rises to 20.2, 24.3, and 21.2 mg g^−1^. At the highest concentration examined (100 mg L^−1^), removal efficiencies drop further to 48.3, 77.0, and 60.1%, whereas *q*_e_ approaches 24.2, 38.5, and 30.1 mg g^−1^ for GO, GQ, and GQS, respectively. This typical behavior reflects the increasing driving force for mass transfer at higher initial Cd(ii) concentrations, which facilitates diffusion from solution to the sorbent surface and allows a larger number of active sites to be occupied, thereby increasing *q*_e_. At the same time, for a fixed amount of sorbent, the total number of available sites is limited; thus, as the number of Cd(ii) ions in solution increases, a progressively smaller fraction of them can be captured, leading to a decline in percentage removal at high initial concentration.^23–28^ The observation that GQ and GQS consistently maintain higher removal efficiencies and capacities than GO over the entire concentration range is fully consistent with the characterization results, which revealed that functionalization of GO with 8-hydroxyquinoline and 8-hydroxyquinoline-5-sulfonic acid introduces additional N- and S-containing chelating motifs and enhances surface reactivity toward Cd(ii).

To gain deeper insight into the adsorption mechanism and surface heterogeneity, the equilibrium data were analyzed using the Langmuir, Freundlich, Temkin, and Sips isotherm models (Fig. 7b–d and Table 4).^43–45^ The Langmuir model provides an excellent description of Cd(ii) adsorption on all three sorbents, with correlation coefficients *R*^2^ = 0.99 and very low *χ*^2^ values (0.08, 0.01, and 0.02 for GO, GQ, and GQS, respectively), indicating that the adsorption process can be approximated as monolayer coverage on a finite number of energetically equivalent sites.^43–45^ The maximum monolayer capacities (*q*_m_) obtained from Langmuir fitting are 26.6, 39.9, and 32.9 mg g^−1^ for GO, GQ, and GQS, respectively, clearly demonstrating that surface functionalization substantially increases the number of effective binding sites. Notably, the Langmuir affinity constants (*k*_L_) are 0.277, 1.082, and 0.261 L mg^−1^ for GO, GQ, and GQS, respectively, showing that GQ not only has the highest capacity but also exhibits the strongest affinity for Cd(ii) among the three sorbents. This trend is mirrored by the Sips model results, where the fitted capacities (*q*_S_ = 26.1, 40.1, and 33.1 mg g^−1^) and affinity constants (*k*_S_ = 0.288, 1.066, and 0.257 L mg^−1^) are essentially identical to their Langmuir counterparts, and the Sips heterogeneity parameters (*m*_S_ = 1.05, 0.99, 0.98) are very close to unity. The latter indicates that surface heterogeneity is limited, consistent with a largely homogeneous distribution of high-affinity sites introduced by the ligand functionalization.^43–45^

**Table 4:** The evaluated parameters of the isotherm models

	GO	GQ	GQS
**Langmuir model**			
*q*_m_ (mg g^−1^)	26.6	39.9	32.9
*k*_L_ (L mg^−1^)	0.27	1.08	0.26
*R* ^2^	0.99	0.99	0.99
*X* ^2^	0.08	0.01	0.02

**Freundlich model**			
1/*n*_F_	0.3	0.3	0.3
*k*_F_ (mg g^−1^) (mg L^−1^)	9.1	20.1	9.8
*R* ^2^	0.88	0.88	0.93
*X* ^2^	3.2	7.8	3.0

**Temkin model**			
*b*_T_ (J mol^−1^)	475.0	331.6	383.5
*A*_T_ (L g^−1^)	3.4	14.9	3.3
*R* ^2^	0.96	0.97	0.99
*X* ^2^	0.7	1.0	0.3

**Sips model**			
*q*_S_ (mg g^− 1^)	26.1	40.1	33.1
*k*_S_ (L mg^− 1^)	0.28	1.06	0.25
*m*_S_	1.05	0.99	0.98
*R* ^2^	0.99	0.99	0.99
*X* ^2^	0.06	0.01	0.01

From an engineering and process-design perspective, the combined interpretation of saturation capacities (*q*_m_, *q*_S_) and affinity constants (*k*_L_, *k*_S_) provides direct guidance for optimizing Cd(ii) removal in industrial wastewater treatment systems. The capacity parameters define the upper sorbent loading limits and enable first-order estimation of sorbent demand for a target effluent concentration, whereas the affinity constants govern uptake efficiency at low-to-moderate Cd(ii) levels and thus determine polishing performance. These equilibrium descriptors are directly applicable to the preliminary design of continuous fixed-bed adsorption systems, where Langmuir/Sips parameters are routinely used to estimate bed capacity, breakthrough behavior, and sorbent replacement frequency.

In this context, the higher *k*_L_ value of GQ (1.082 L mg^−1^) highlights its suitability for treating dilute Cd(ii) effluents or for use as a final polishing stage to meet stringent discharge limits, while GO and GQS are more appropriate for moderate-removal stages where adsorption affinity is less critical. The decline in removal efficiency observed at higher initial concentrations under a fixed sorbent dose further indicates that industrial optimization should rely on dose adjustment, staged adsorption (*e.g.*, series contactors), or continuous fixed-bed operation to maintain high treatment efficiency under elevated Cd(ii) loadings. Collectively, the combined capacity–affinity ranking (GQ > GQS > GO) supports the selection of GQ for compact, high-performance adsorption units, while GQS and GO remain viable options when simpler treatment objectives and economic considerations prevail. Importantly, the batch-derived kinetic and equilibrium parameters reported here represent intrinsic sorbent–adsorbate properties that are widely used as the foundation for preliminary process design, modeling, and subsequent scale-up to continuous adsorption systems.

The Freundlich and Temkin models provide complementary perspectives on surface heterogeneity and energetic interactions.^43–45^ Freundlich fits yield correlation coefficients of 0.88, 0.88, and 0.93 for GO, GQ, and GQS, respectively, markedly lower than those of Langmuir/Sips, but still informative. The Freundlich constants *k*_F_ (9.1, 20.1, and 9.8) and 1/*n*_F_ (0.3 for all sorbents) indicate highly favorable adsorption (1/*n*_F_ < 1) and again highlight the superior sorption intensity of GQ, in line with its higher population of chelating sites.^20–22^ Temkin analysis, which assumes a linear decrease in the heat of adsorption with coverage, also shows good agreement with the experimental data (*R*^2^ = 0.96–0.99). The Temkin constants *b*_T_ (475.0, 331.6, and 383.5 J mol^−1^) and *A*_T_ (3.4, 14.9, and 3.3 L g^−1^) suggest relatively strong sorbent–Cd(ii) interactions, with GQ displaying the highest AT value, again reflecting its stronger binding propensity.

Overall, the concentration-dependent uptake trends and multi-model isotherm fitting collectively confirm that Cd(ii) adsorption onto GO, GQ, and GQS is favorable and well represented by Langmuir/Sips behavior, with functionalization markedly enhancing both capacity and affinity. Therefore, *q*_m_ values should be interpreted as design benchmarks derived from equilibrium modeling rather than direct operational capacities under complex wastewater conditions. Importantly, these equilibrium descriptors are not only mechanistic indicators but also practical design handles for wastewater treatment, enabling rational selection between high-affinity polishing (favoring GQ) and broader, cost-driven treatment stages (where GQS/GO may remain viable) depending on influent variability and discharge targets. This equilibrium behavior is consistent with numerous Cd(ii) adsorption studies on graphene-based sorbents and related composites, where Langmuir/Sips-type fitting is frequently reported and attributed to site-specific binding on accessible functional groups. Illustrative examples include Graphene oxide (GO),^23^ amine-functionalized GO materials,^24^ A GO/paper hybrid prepared from waste fibers,^25^ magnetic GO composites—including GO-tryptophan (MGO-Trp),^26^ α-Fe_2_O_3_/GO,^27^ few-layered magnetic graphene oxide (FLMGO),^28^ and PAN = Fe_3_O_4_@GO,^29^ and cyclodextrin-GO hybrids.^31^

The experimentally observed affinity order GQ > GQS > GO can be rationalized by recognizing that all three sorbents share the same graphene oxide backbone but differ markedly in the strength, density, and accessibility of surface complexation sites introduced during functionalization. Pristine graphene oxide (GO), characterized by a broad XRD (002) reflection at ∼10° (2*θ*) (Fig. 1), a relatively high BET surface area (75 m^2^ g^−1^) (Table 1), and abundant oxygen-containing functionalities (FTIR bands of –OH at 3200–3500 cm^−1^, C=O at ∼1720 cm^−1^, and C–O at 1220–1050 cm^−1^) (Fig. 2), provides a surface dominated by electrostatic interactions and weak coordination sites. SEM–EDS confirms a C/O-rich composition (Fig. 3), while the moderately negative zeta potential (−21 mV) (Table 2) favors Cd^2+^ attraction. However, these oxygenated groups bind Cd(ii) mainly through monodentate O-donor interactions, which are relatively weak and non-specific.^30,31^ As a result, GO exhibits the smallest pseudo-second-order rate constant (*k*_2_ = 0.005 min^−1^) (Table 3) and the lowest Langmuir affinity constant (*k*_L_ = 0.28 L mg^−1^) (Table 4), reflecting its limited ability to form strong inner-sphere complexes.

Functionalization of GO with 8-hydroxyquinoline (8-HQ) yields GQ, in which a high density of well-defined N,O-chelating sites is introduced.^20,21^ FTIR spectra show distinct C=N (1570 cm^−1^) and C–N/aromatic C–O (1280 cm^−1^) bands (Fig. 2), while EDS confirms nitrogen incorporation (Fig. 3). XRD patterns indicate preservation of the layered framework with increased disorder (Fig. 1), and BET analysis shows that mesoporosity is maintained (mode pore diameter = 2.94 nm) despite a reduction in surface area to 35 m^2^ g^−1^ (Table 1). The slightly less negative zeta potential (−15 mV) (Table 2) facilitates closer approach of Cd(ii) ions to the chelation sites. Importantly, 8-HQ ligands coordinate Cd(ii) bidentately *via* the quinolinic nitrogen and adjacent phenolic oxygen, forming stable five-membered chelate rings.^20,21^ This strong inner-sphere complexation, combined with preserved pore accessibility and favorable electrostatic conditions, explains why GQ exhibits the highest monolayer sorption capacity (*q*_m_ = 40 mg g^−1^), the strongest affinity constant (*k*_L_ = 1.08 L mg^−1^) (Table 4), the fastest kinetic response (*k*_2_ = 0.010 min^−1^), and the highest initial sorption rate (*h* = 5.9 mg g^−1^ h^−1^) (Table 3).

In contrast, GQS is obtained by activating GO with 8-hydroxyquinoline-5-sulfonic acid, which preserves the N,O-chelation motif of 8-HQ but introduces a strongly electron-withdrawing sulfonic group (–SO_3_H) at the 5-position of the quinoline ring.^48,49^ FTIR spectra display intense S=O stretching vibrations in the range 1120–1180 cm^−1^ (Fig. 2), and sulfur incorporation is confirmed by EDS (Fig. 3). XRD analysis reveals sharper crystalline features compared with GQ (Fig. 1), while BET measurements show a further decrease in surface area to 23 m^2^ g^−1^ (Table 1), indicating reduced textural accessibility. This loss of accessible surface area likely limits diffusion and exposure of internal chelation sites, particularly under equilibrium loading conditions. Although GQS exhibits a more negative zeta potential (−17 mV) (Table 2), which enhances electrostatic attraction toward Cd^2+^, DLS results indicate increased aggregation heterogeneity (Table 2). Moreover, the sulfonate substituent introduces additional steric bulk and reduces ligand basicity, partially weakening Cd-ligand coordination and restricting access to neighboring N-donor sites.^48,49^ Consequently, despite benefiting from mixed N,O-donor chelation and electrostatic contributions, the combined steric and electronic effects imposed by sulfonation reduce the effective accessibility and binding strength of the chelating sites. This balance of factors results in an overall adsorption performance intermediate between GQ and GO, as reflected by *k*_2_ = 0.007 min^−1^ (Table 3), *q*_m_ = 33 mg g^−1^, and *k*_L_ = 0.26 L mg^−1^ (Table 4).

When the activation pathways are considered collectively (GO → GQ *via* 8-HQ and GO → GQS *via* 8-HQS), the affinity trend GQ > GQS > GO emerges naturally as a direct consequence of (i) the superior intrinsic chelation strength of 8-HQ relative to its sulfonated analogue, (ii) differences in the density and accessibility of grafted chelating sites, and (iii) the interplay between electrostatic attraction and true inner-sphere complexation. Overall, the progression from weak oxygen-based binding sites in GO, through chemically diverse but partially sterically constrained sites in GQS, to optimally accessible and strongly chelating quinoline sites in GQ provides a coherent structure–function framework for understanding the observed adsorption behavior.

A comparison with previously reported Cd(ii) sorbents (Table 5) illustrates that the adsorption capacities achieved in this study—26.6 mg g^−1^ for GO, 39.9 mg g^−1^ for GQ, and 32.9 mg g^−1^ for GQS—are well within the range observed for many graphene-based and composite adsorbents. Several materials reported in the literature exhibit moderate capacities comparable to those of the present sorbents, including amine-functionalized GO (10.04 mg g^−1^),^24^ GO modified with waste newspaper (31.35 mg g^−1^),^25^ CuO-modified ceramic membranes (12.4 mg g^−1^),^38^ pectin hydrogel/Fe_3_O_4_/bentonite (35.5 mg g^−1^),^39^ magnetite–chitosan composites (18.67 mg g^−1^),^53^ and Algerian sheep horn powder (23.8 mg g^−1^).^54^ Although some advanced systems such as MBT-modified GO (79.7 mg g^−1^),^32^ GO aerogels (108.7 mg g^−1^),^35^ sulfidized nanoscale zero-valent iron (126.9 mg g^−1^),^36^ and Ag-MOF/CSC composite sponges (193.3 mg g^−1^)^40^ exhibit higher capacities, their preparation often involves energy-intensive processing, multistep chemical modification, or costly reagents. Accordingly, capacity comparisons should be interpreted alongside practical descriptors such as synthesis complexity, operating pH/temperature, contact time, regeneration behavior, and performance in competitive ionic matrices, which collectively govern real-world feasibility. While several modified graphene oxide–based sorbents achieve higher Cd(ii) uptake, these systems frequently rely on complex synthesis routes or operate under non-ambient conditions. In contrast, GQ and GQS combine a simple ligand-functionalization strategy (without inorganic nanoparticle loading or polymeric scaffolds), mild operating conditions (pH 6, 25 °C), and a chemically interpretable chelation mechanism dominated by quinoline N,O-donor coordination, which is advantageous for rational optimization and scale-relevant implementation. Moreover, the selectivity of the present sorbents is supported by real raffinate testing, where Cd(ii) exhibits substantially higher affinity than coexisting ions as reflected by distribution coefficients (*K*_d_) (Table S5), thereby strengthening the practical impact beyond single-solute laboratory systems. Therefore, the present comparison benchmarks sorbent performance not only in terms of maximum adsorption capacity but also with respect to synthetic simplicity, operational requirements, selectivity, and regeneration potential—criteria that are critical for translation to industrial wastewater treatment applications.

**Table 5:** Comparison of equilibrium adsorption capacities (*q*_e_, mg g^−1^) of various sorbents for Cd(ii) removal from aqueous solutions under reported experimental conditions

Sorbent type	*C*_0_, mg L^−1^	Temp	pH	Time, min	*q*_e_, (mg g^−1^)	Ref.
Amine-functionalized GO materials	10–300	25	5	240	10.04	24
Graphene oxide modified waste newspaper	—	25	6	720	31.35	25
GO modified 2-mercapto benzothiazole (MBT)	10–100	25	7	120	79.7	32
Graphene oxide aerogel (GOA)	—	25	6	120	108.7	35
Sulfidized nanoscale zero-valent iron	50–400	25	6	2880	126.9	36
Magnetic iron-modified calcium silicate hydrate	—	25	4	300	16.39	37
CuO-modified ceramic membrane	6.3–44.5	25	4	120	12.4	38
Pectin hydrogel/Fe3O4/Bentonite	100–300	25	4.7	45	35.5	39
Ag-MOF/CSC composite sponge	—	25	5	60	193.3	40
Magnetite–Chitosan composite	0–200	25	5	10	18.67	53
Algerian sheep horns powder (BKASH)	20–100	25	6	30	23.8	54
GO	10–100	25	6	240	26.6	PW
GQ					39.9	
GQS					32.9	

#### 3.2.5 Impact of temperature and thermodynamic investigation

**Fig. 8:**
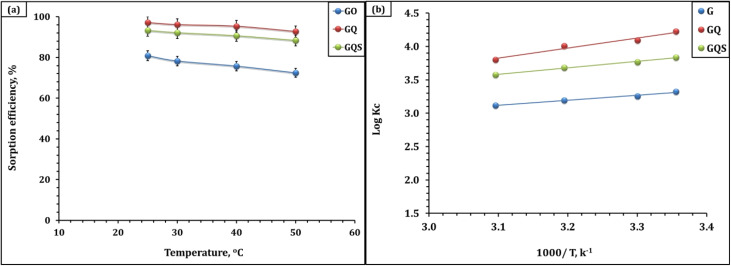
Effect of temperature on the adsorption efficiency of Cd(ii) ions (a), van't Hoff plot (b). Experimental conditions: time = 240 min, pH = 5.98, adsorbent dose = 2.0 g L^−1^, initial concentration 50 mg L^−1^.

The influence of temperature on Cd(ii) adsorption by GO, GQ, and GQS was evaluated in the range 25–50 °C at fixed initial concentration (50 mg L^−1^), sorbent dose (2.0 g L^−1^), contact time (240 min), and optimized pH (Fig. 8a). The temperature-dependent data reveal a clear, systematic decline in both removal efficiency and equilibrium uptake (*q*_*t*_) for all three adsorbents—GO, GQ, and GQS—as the solution temperature rises from 25 °C to 50 °C. At 25 °C, GO removes 80.8% of Cd(ii) ions (*q*_*t*_ = 20.2 mg g^−1^), GQ achieves 97.1% removal (*q*_*t*_ = 24.3 mg g^−1^), and GQS 93.2% (*q*_*t*_ = 23.3 mg g^−1^). By 50 °C, these values fall to 72.4% (18.1 mg g^−1^), 92.7% (23.2 mg g^−1^), and 88.3% (22.1 mg g^−1^), respectively. This inverse relationship between temperature and adsorption capacity typifies an exothermic process: as thermal energy increases, the equilibrium shifts toward desorption, reducing the fraction of Cd(ii) bound to the adsorbent surface.^25–27^ At elevated temperatures, enhanced molecular motion weakens the adsorbate–adsorbent interactions and promotes partial disruption of surface complexes, thereby favoring Cd(ii) release back into solution. The fact that GQ and GQS consistently maintain higher removal efficiencies than GO at all temperatures is fully consistent with their richer populations of N,O-donor chelating and sulfonated sites, as established in the characterization and isotherm sections.

Thermodynamic parameters derived from van't Hoff analysis provide further insight into the nature of Cd(ii) binding on these sorbents (Fig. 8b and Table 6).^46,47^ The thermodynamic parameters further substantiate the exothermic and spontaneous nature of the adsorption. For GO, the standard enthalpy change (Δ*H*°) is −14.4 kJ mol^−1^, and the standard entropy change (Δ*S*°) is +15.2 J mol^−1^ K^−1^. The negative Δ*H*° confirms that heat is released upon Cd(ii) binding, while the positive Δ*S*° suggests that the overall system—adsorbent plus solution—becomes more disordered. This entropy gain likely arises from the release of structured water molecules from the GO surface as Cd(ii) species occupy adsorption sites.^25–27^ As temperature increases, this enthalpy-driven binding becomes less favorable relative to thermal agitation, explaining the gradual decrease in uptake despite a positive entropy contribution. The corresponding Gibbs free energies (Δ*G*°) remain negative and only slightly more favorable at higher temperature (from −19.0 kJ mol^−1^ at 25 °C to −19.3 kJ mol^−1^ at 50 °C), consistent with a predominantly physical adsorption mechanism aided by an entropic driving force.^25–27^

**Table 6:** The values of the thermodynamic parameters

	Δ*G*° (kJ mol^−1^)				Δ*H*°	Δ*S*°
	25 °C	30 °C	40 °C	50 °C	(kJ mol^−1^)	(J mol^−1^ K^−1^)
GO	−19.0	−18.9	−19.1	−19.3	−14.4	15.2
GQ	−24.1	−23.7	−24.0	−23.5	−28.8	−15.9
GQS	−21.9	−21.8	−22.1	−22.1	−18.5	11.4

GQ exhibits a larger exothermicity (Δ*H*° = −28.8 kJ mol^−1^) but a negative entropy change (Δ*S*° = −15.9 J mol^−1^ K^−1^). The magnitude of Δ*H*° hints at stronger interactions—possibly a combination of van der Waals forces, π–π stacking, and some degree of chemisorption—between the quinoline moiety and the target metal ion. The negative Δ*S*° indicates that the adsorbate–adsorbent assembly is more ordered than the separate components, perhaps due to the formation of well-defined Cd(ii)–quinoline complexes at the surface.^46,47^ In this case, increasing temperature disrupts these ordered surface complexes, directly reducing adsorption capacity and underscoring the dominance of enthalpy-controlled binding. Despite the decrease in randomness, Δ*G*° remains substantially negative across the studied temperature range (from −24.1 kJ mol^−1^ at 25 °C to −23.5 kJ mol^−1^ at 50 °C), confirming a spontaneous process whose driving force is dominated by the exothermic enthalpy term. For GQS, Δ*H*° is −18.5 kJ mol^−1^ and Δ*S*° is +11.4 J mol^−1^ K^−1^, again pointing to exothermic adsorption coupled with an increase in disorder.^46,47^ Sulfonate functionalization appears to increase the release of hydration water or to disrupt solution structure even more effectively than GO alone, yielding a positive entropy contribution.^48,49^ Nevertheless, the negative Δ*H*° ensures that adsorption remains enthalpy-dominated, and higher temperatures progressively weaken electrostatic and coordination interactions, leading to reduced uptake. The Δ*G*° values (−21.9 kJ mol^−1^ at 25 °C to −22.1 kJ mol^−1^ at 50 °C) are intermediate between those of GO and GQ, reflecting a balance of moderately strong enthalpic interactions and favorable entropy gain.

Taken together, the temperature-dependent adsorption efficiencies and thermodynamic parameters indicate that all three sorbents remove Cd(ii) through spontaneous and exothermic processes. GO and GQS behave predominantly as physisorptive materials, driven by favorable entropy changes and weaker intermolecular interactions, whereas GQ exhibits more ordered and energetically stronger binding, consistent with partial chemisorptive character. The systematic decline in adsorption capacity with increasing temperature reflects the progressive destabilization of surface-bound Cd(ii) complexes and confirms that lower operating temperatures favor retention of Cd(ii) on the sorbent surface. Moreover, the magnitudes and signs of Δ*G*°, Δ*H*°, and Δ*S*° clearly differentiate the interaction mechanisms operating across the three materials. Similar thermodynamic behavior has been widely reported for other Cd(ii) sorbents, where adsorption consistently proceeds spontaneously (Δ*G*° < 0) and exothermically. Examples include graphene oxide (GO),^23^ GO/paper hybrids derived from waste fibers,^25^ magnetic GO-tryptophan composites (MGO-Trp),^26^ and α-Fe_2_O_3_/GO systems,^27^ all of which exhibit thermodynamic signatures comparable to those observed here. Collectively, these findings demonstrate that diverse sorbent families share common thermodynamic trends in Cd(ii) uptake, reinforcing the robustness and validity of the thermodynamic conclusions drawn from the present study.

### 3.3 Proposed adsorption mechanisms

Understanding the adsorption mechanism is essential for elucidating how the structural and chemical features of GO, GQ, and GQS govern their ability to capture Cd(ii) ions. Mechanistic insights not only validate the experimental findings but also provide a rational framework for designing next-generation graphene-based adsorbents with enhanced selectivity, capacity, and reusability. In the broader context of graphene oxide research, numerous studies have revealed that GO and its functionalized derivatives interact with heavy metal ions through a diverse yet coherent set of synergistic processes. Across systems such as amino-functionalized GO,^24^ magnetic GO-polymer hybrids,^29^ nitrogen- and sulfur-functionalized GO,^32,55^ magnetic few-layered GO,^28^ pristine GO and GO-based wastewater adsorbents,^56^ cyclodextrin-modified GO,^31,34^ magnetic GO-cellulose composites,^30^ GO-based Cd(ii)/Cu(ii) sorbents,^23^ redox-active modified GO,^57^ Fe-Zr-AlO_*x*_/GO-chitosan aerogels,^58^ COF-decorated GO,^59^ and halogen/sulfur-functionalized carbon materials,^60^ heavy-metal removal typically proceeds through electrostatic attraction, inner-sphere complexation, chelation, ion exchange, π–cation and π–π interactions, host–guest inclusion, hydrogen bonding, and in some cases redox-assisted pathways. These functional modifications introduce diverse high-affinity donor sites (N, S, aromatic heterocycles, metal oxides), effectively transforming GO from a primarily oxygen-based adsorbent into a multimodal chelating platform capable of strong, cooperative metal binding across a wide range of heavy metals.^24,28,29,31,54–61^

In the present work, for the three sorbents examined, the adsorption of Cd(ii) begins with electrostatic attraction between hydrated Cd^2+^ ions and the negatively charged surfaces of GO, GQ, and GQS. This mechanism is widely reported in GO-based Cd(ii) and Cu(ii) removal systems, where deprotonated –COO^−^ and phenolic –O^−^ groups provide the initial driving force for metal approach.^23,30,56^ Zeta potential measurements in this work confirm similarly negative surfaces, which facilitate the migration and accumulation of Cd^2+^ at the solid–liquid interface, consistent with observations in magnetic GO–cellulose composites^30^ and GO/paper hybrids.^57^ As Cd^2+^ approaches the surface, partial dehydration of its hydration shell occurs—a step documented in cyclodextrin–GO systems^31,34^ and Fe–Zr–AlO_*x*_/GO–chitosan aerogels,^58^ and reflected here by the positive Δ*S*° values.

Following electrostatic enrichment, Cd(ii) binding proceeds *via* surface complexation, chelation, and ion exchange. In GO, oxygen-containing groups such as –COOH, –OH, epoxy, and carbonyl units serve as the primary coordination sites, forming mono- and bidentate complexes, a mechanism identical to those reported for GO–cellulose hybrids,^30^ and GO–Fe_3_O_4_ systems,^28^ pristine GO.^56^ In GQ, the introduction of 8-hydroxyquinoline creates N, O-bidentate chelation sites, which form stable five-membered chelate rings with Cd(ii). Similar N, O-chelation has been reported in amino-functionalized GO,^24^ nitrogen-rich graphene adsorbents,^23,32^ and heterocycle-modified GO,^55^ all of which exhibit enhanced affinity due to strong inner-sphere coordination. For GQS, grafting 8-hydroxyquinoline-5-sulfonic acid introduces both N, O-chelating sites and a sulfonate group (–SO_3_^−^). Sulfonate-enhanced electrostatic attraction has been previously documented in sulfur-functionalized graphene frameworks^60^ and sulfur-rich GO composites.^32^ The electron-withdrawing nature of –SO_3_^−^ slightly decreases the basicity of the quinoline N, O donor pair, producing affinity values intermediate between GQ and GO—an effect similarly observed in mixed N/S or S-containing GO adsorbents.^55,60^

Additional secondary interactions complement these primary mechanisms. Hydrogen bonding contributes to stabilization in systems such as GO–cellulose,^30^ GO–chitosan aerogels,^58^ and CD/GO composites.^31,34^ van der Waals interactions and pore filling are supported by the preserved mesoporosity observed in this work and corroborated by analogous findings in COF–GO hybrids^59^ and porous GO-based membranes.^23^ Intraparticle diffusion and boundary-layer effects, indicated by multi-linear Weber–Morris plots, follow the same sequence reported for magnetic GO sorbents^28,29^ and polymer-assisted GO adsorbents.^23^ Furthermore, π–cation and π–π interactions—documented in aromatic GO composites^55,59^—likely contribute to stabilization of Cd(ii) near quinoline rings in GQ and GQS.

Thermodynamic parameters reinforce these mechanistic interpretations. The negative Δ*G*° values obtained here indicate spontaneous adsorption, consistent with reports for pristine GO,^32^ GO/paper hybrid systems,^57^ magnetic GO-tryptophan composites (MGO–Trp),^58^ and α-Fe_2_O_3_/GO sorbents.^59^ The exothermic nature of adsorption, confirmed by decreasing capacity with increasing temperature, matches behaviors reported in Cd(ii) adsorption on GO-based materials.^23,30,34^ Positive Δ*S*° values—reflecting increased interfacial disorder due to water displacement during complexation—are similarly observed in cyclodextrin-GO,^31,34^ amino-GO,^24^ and COF-GO systems.^59^

**Fig. 9:**
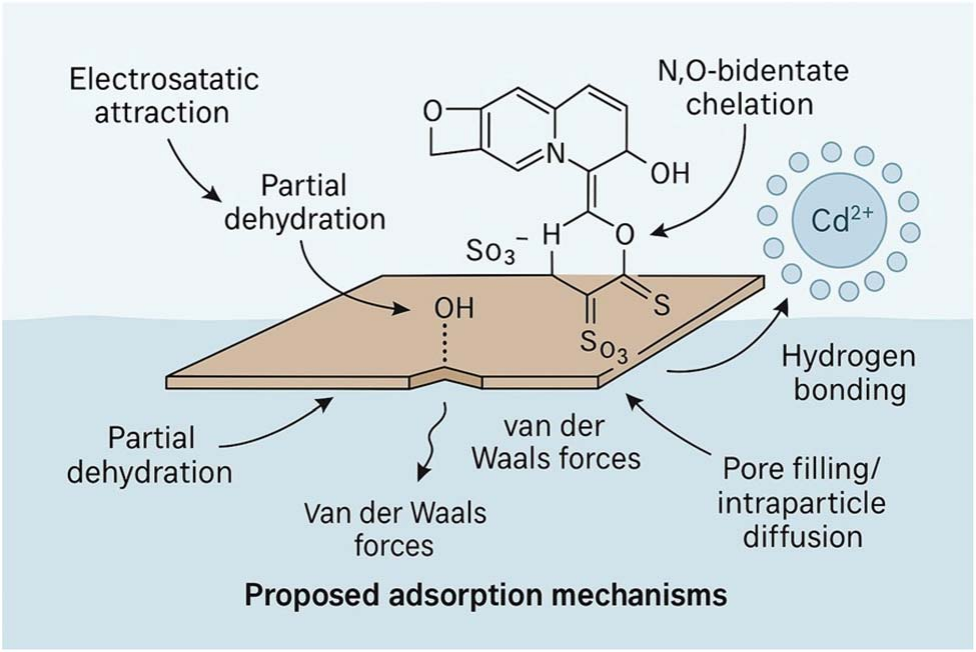
Schematic illustration of the proposed interaction mechanism for Cd(ii) adsorption.

Collectively, adsorption of Cd(ii) onto GO, GQ, and GQS in this study occurs through a synergistic combination of: (i) electrostatic attraction (consistent with ref. 23, 30, 56 and 57); (ii) partial dehydration of Cd^2+^ (as observed in ref. 31, 34, and 58); (iii) inner-sphere coordination to oxygen, nitrogen, and sulfur donor atoms (documented in ref. 23, 24, 28, 30, 32, 55 and 56); (iv) chelation *via* quinoline-based N,O or N,O/S ligands (supported by ref. 23, 24, 31, 32 and 55); (v) Secondary interactions including hydrogen bonding, van der Waals forces, π–cation, and π–π interactions (supported by ref. 30, 31, 34, 59 and 60); and (vi) Pore filling and intraparticle diffusion within mesoporous networks (consistent with ref. 23, 24, 29 and 58). These unified mechanisms align with the broad mechanistic landscape reported for functionalized GO sorbents in the literature and fully explain the kinetic, isotherm, and thermodynamic behavior observed for GO, GQ, and GQS in the present work.^24,28,29,31,54–61^ A schematic illustration of the proposed adsorption pathways, integrating all primary and secondary interactions discussed above, is shown in Fig. 9.

### 3.4 Desorption investigation and application in waste solution (case study)

The desorption behavior of Cd(ii) from the loaded GQ sorbent was evaluated using three mineral acids—1.0 M HCl, 1.0 M HNO_3_, and 1.0 M H_2_SO_4_—in order to identify an effective regenerating agent and gain insight into the binding strength and reversibility of the surface complexes (Table S4). The results clearly show that all three eluents are capable of stripping a substantial fraction of Cd(ii), but with markedly different efficiencies: 1.0 M HCl achieves the highest desorption efficiency (93.6%), followed by 1.0 M HNO_3_ (84.6%) and 1.0 M H_2_SO_4_ (80.7%). The high overall desorption values confirm that Cd(ii) binding, while strong, is not irreversible and that the sorbent can, in principle, be regenerated and reused.^51,52^ From a practical standpoint, the = 94% desorption efficiency with 1.0 M HCl suggests that this medium is a promising regenerant for recycling the sorbent in multiple adsorption–desorption cycles, while also providing a convenient route to recover Cd(ii) in a concentrated eluate. Although extended multi-cycle regeneration was not evaluated in this study, the desorption experiments were designed as single-step eluent screening to identify an effective stripping medium. Potential ligand leaching or structural changes under repeated strong-acid regeneration were beyond the scope of the present work.

To further assess practical applicability, a real industrial raffinate solution collected from the Electroplate Co. (10th of Ramadan Industrial Zone, Egypt), containing 120 mg L^−1^ Cd(ii) together with seven coexisting species, was treated under the same optimized conditions established for single-component experiments. The initial (*C*_0_) and equilibrium (*C*_e_) concentrations, along with the corresponding removal efficiencies, are summarized in Table S5. Notably, GQ achieved 94.2% removal of Cd(ii) while exhibiting only limited uptake of competing ions. Specifically, the removal efficiencies of V(v) and Si(iv) were 20.0% and 23.3%, respectively, while Cr(iii) and Ca(ii) were removed at 11.3% and 19.8%. Monovalent species showed even weaker interaction, with Na(i) and Cl^−^ removal limited to 15.0% and 8.8%, respectively. To quantitatively substantiate selectivity beyond percentage removal, distribution coefficients (*K*_d_) were calculated for Cd(ii) and all coexisting ions (Table S5). Cd(ii) exhibited a markedly higher *K*_d_ value (8.07 L g^−1^) compared with the competing species (0.05–0.15 L g^−1^), corresponding to an approximately one–two orders of magnitude difference. This pronounced contrast confirms preferential Cd(ii) uptake and minimal competitive interference under realistic multi-component conditions, highlighting the strong affinity of the quinoline-functionalized surface toward Cd(ii) relative to both transition-metal cations and common light-metal and anionic species. Importantly, this behavior is consistent with the pH-dependent adsorption trends (Section 3.2.1), where pH = 6 was identified as optimal due to concurrent surface deprotonation and Cd^2+^ predominance, thereby favoring electrostatic attraction and chelation. The real-sample experiment thus demonstrates that the same pH-guided adsorption mechanism remains effective even in a complex ionic matrix.

Taken together, these findings demonstrate that the developed sorbent combines high regenerability—enabled by a simple acid wash restoring more than 90% of Cd(ii) binding capacity—with pronounced selectivity under realistic wastewater conditions. While extended multi-cycle reuse was not examined in the present work, the high desorption efficiency, preserved adsorption performance in real wastewater, and absence of harsh regeneration requirements collectively suggest good chemical stability and reuse potential, consistent with previously reported graphene oxide–based adsorbents. The observed performance can be attributed to the optimized chelating functionality of the GQ surface, favorable size and charge complementarity for Cd(ii), and effective competitive exclusion of coexisting ions under the selected pH and ionic strength conditions. From an application perspective, such behavior is promising for cost-effective treatment of cadmium-bearing waste streams, minimizing secondary contamination by co-adsorbed species and enabling straightforward recovery of Cd(ii) for either safe disposal or resource reclamation. It should be noted that the maximum adsorption capacities derived from equilibrium isotherm modeling represent idealized upper limits under controlled laboratory conditions; in practical wastewater treatment scenarios, effective performance is governed by matrix composition, coexisting ions, operating pH, and regeneration strategy. Nevertheless, the high Cd(ii) removal efficiency achieved here under realistic conditions clearly confirms the practical relevance of the developed sorbents.

## 4 Conclusion

This study demonstrates that targeted chemical functionalization of graphene oxide with quinoline-based ligands markedly enhances its adsorption performance toward Cd(ii), highlighting the decisive role of ligand chemistry over surface area alone. Although pristine GO exhibited the highest specific surface area (75.3 m^2^ g^−1^), its adsorption capacity was comparatively limited due to the predominance of weak oxygen-based interactions. In contrast, functionalization with 8-hydroxyquinoline (GQ) and 8-hydroxyquinoline-5-sulfonic acid (GQS) introduced well-defined N,O- and N,O/S-donor sites, respectively, significantly strengthening surface complexation despite reduced surface areas (34.7 and 23.4 m^2^ g^−1^). Batch adsorption experiments revealed strongly pH-dependent behavior, with optimal Cd(ii) uptake at pH = 6 and an optimal sorbent dosage of 2.0 g L^−1^. Kinetic investigations showed rapid uptake, with more than 90% of equilibrium adsorption achieved within 120–180 min, and were best described by the pseudo-second-order model (*R*^2^ > 0.998), indicating adsorption dominated by site-specific interactions. Equilibrium data were well fitted by the Langmuir and Sips models, yielding maximum adsorption capacities of 39.9 mg g^−1^ for GQ, 32.9 mg g^−1^ for GQS, and 26.6 mg g^−1^ for GO, consistent with the accessibility and strength of their respective coordination sites. Thermodynamic analysis (Δ*H*° < 0 and Δ*G*° < 0, with Δ*S*° dependent on surface functionalization) confirmed a spontaneous and exothermic adsorption process, governed by chelation-driven inner-sphere complexation and interfacial dehydration. Mechanistic interpretation indicates that Cd(ii) uptake proceeds through a synergistic combination of electrostatic attraction, chelation, inner-sphere complexation, hydrogen bonding, van der Waals interactions, and mesopore-assisted diffusion. Among the investigated sorbents, GQ exhibited the highest overall affinity owing to the optimal accessibility of its N,O-bidentate chelating sites, whereas GQS benefited from additional sulfonate-induced electrostatic attraction but showed slightly constrained site accessibility. Pristine GO relied primarily on weaker oxygen-based interactions. Desorption experiments confirmed excellent regenerability, with 1.0 M HCl achieving up to 93.6% Cd(ii) recovery. Importantly, application to a real industrial raffinate demonstrated 94.2% Cd(ii) removal with minimal co-ion uptake. Selectivity under competitive conditions was quantitatively supported by distribution coefficients, with Cd(ii) exhibiting *K*_d_ values one to two orders of magnitude higher than those of coexisting ions. The optimal adsorption pH (=6), close to that of many industrial effluents, further supports practical applicability with minimal pH adjustment. Overall, this work establishes quinoline-functionalized graphene oxide as an efficient, selective, and regenerable platform for Cd(ii) remediation in complex aqueous matrices. Compared with many reported modified GO systems that rely on multicomponent or nanoparticle-based architectures, the present ligand-driven functionalization strategy offers a simpler, chemically interpretable, and tunable route for enhancing selectivity and binding strength. These features make GQ and GQS promising candidates for scalable adsorption-based treatment schemes targeting cadmium-contaminated industrial wastewater.

## Conflicts of interest

The authors of this article would like to confirm that all of them have no conflict of interests with any organization or any person and the funding body is listed.

## Supplementary Material

RA-016-D6RA00200E-s001

## Data Availability

The datasets used and/or analyzed during the current study are available from the corresponding author on reasonable request. Supplementary information (SI): Fig. S1: BET isotherm of graphene oxide (GO), GO functionalized with 8-hydroxyquinoline (GQ), and GO functionalized with 8-hydroxyquinoline-5-sulfonic acid (GQS) sorbents; Fig. S2: DLS of graphene oxide (GO), GO functionalized with 8-hydroxyquinoline (GQ), and GO functionalized with 8-hydroxyquinoline-5-sulfonic acid (GQS) sorbents; Fig. S3: expected aqueous speciation of metal ion concentration (50 mg L^−1^) for cadmium as a function of pH using Medusa/Hydra program; Fig. S4: intraparticle diffusion (IPD) model plots for Cd(ii) adsorption. Experimental conditions: initial ion concentration = 50 mg L^−1^, pH = 5.01, dose = 0.5 g L^−1^, temperature = 25 °C; Fig. S5: separation factor (*R*_1_) for Cd(ii) adsorption process using GO, GQ, GQS sorbents; Table S1: kinetic, isotherm, and thermodynamics equations for and Cd(ii) sorption process; Table S2: the relationship between Cd(ii) adsorption capacity and sorbent dose under the following conditions: room temperature, a pH of 6.0, a starting concentration of 50 mg L^−1^, reaction time of 240 min; Table S3: the values of Morris–Weber model parameters; Table S4: Cd(ii) desorption from loaded sorbent using different solutions (2.0 g L^−1^, room temperature; 120 min); Table S5: initial concentrations of constituents in raffinate wastewater, along with calculated removal efficiency (%), and distribution coefficient (*k*_d_) after treatment with GQ sorbent. See DOI: https://doi.org/10.1039/d6ra00200e.
