# Supplementary material for: Quinoline-functionalized graphene oxide for enhanced cadmium removal: synthesis, characterization, and mechanistic insights
Source: RSC Adv. 2026 Mar 13;16(16):13960–81. doi: 10.1039/d6ra00200e (PMC12983473; doi:10.1039/d6ra00200e)
Supplement: RA-016-D6RA00200E-s001 [file RA-016-D6RA00200E-s001.pdf]

Supplementary Information (SI) for RSC Advances.
This journal is © The Royal Society of Chemistry 2026

Quinoline-Functionalized Graphene Oxide for Enhanced Cadmium Removal: Synthesis, Characterization, and Mechanistic Insights

Huda Ammar*[1], Mohamed F. Kamel [1], Ahmed M. Masoud *[1], Entsar H. Taha[2], Adel A. El-Zahhar[3], Majed M. Alghamdi[3], Mohamed H. Taha[1]

[1] *Nuclear Materials Authority, P.O. Box 530, El Maddi, Cairo, Egypt*
[2] *Department of Plant Protection, Faculty of Agriculture, Ain Shams University, Cairo, Egypt*
[3] *Department of Chemistry, Faculty of Science, King Khalid University, P.O.Box 9004, Abha 61413, Saudi Arabia*

* Corresponding author: ammar.huda@yahoo.com , chemmaso010@hotmail.com

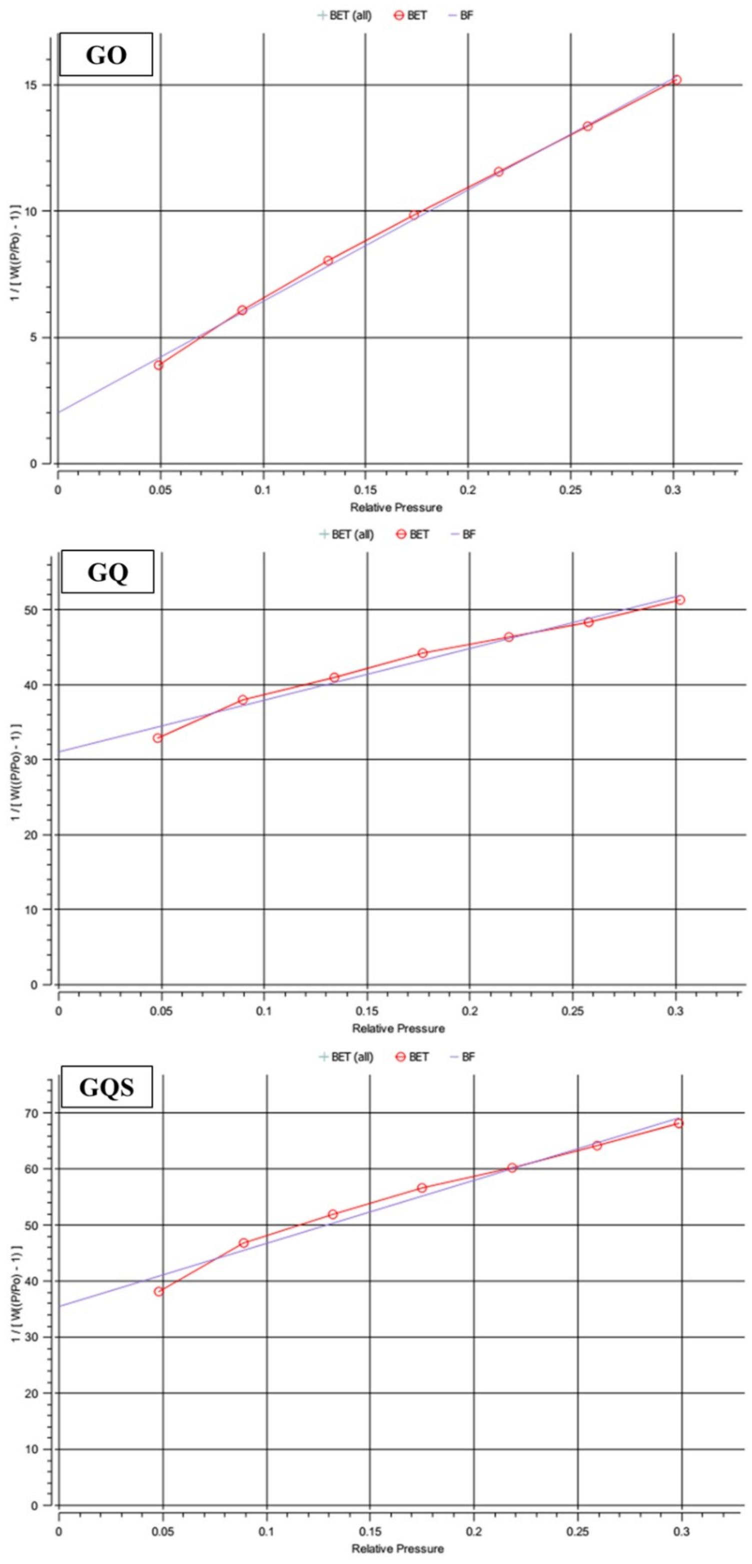

Figure S1: *BET isotherm of graphene oxide (GO), GO functionalized with 8-hydroxyquinoline (GQ), and GO functionalized with 8-hydroxyquinoline-5-sulfonic acid (GQS) sorbents.*

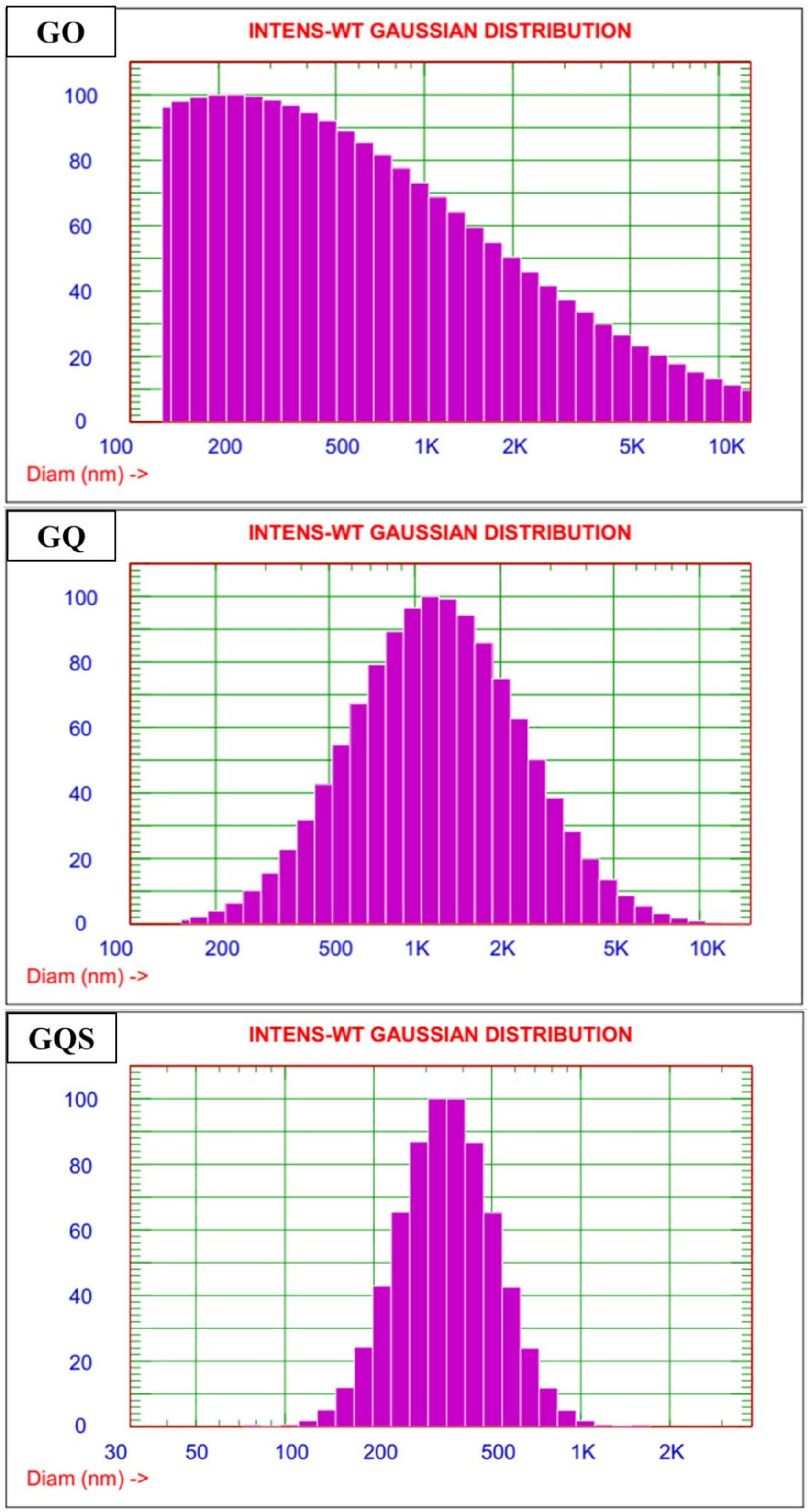

Figure S2: *DLS of graphene oxide (GO), GO functionalized with 8-hydroxyquinoline (GQ), and GO functionalized with 8-hydroxyquinoline-5-sulfonic acid (GQS) sorbents.*

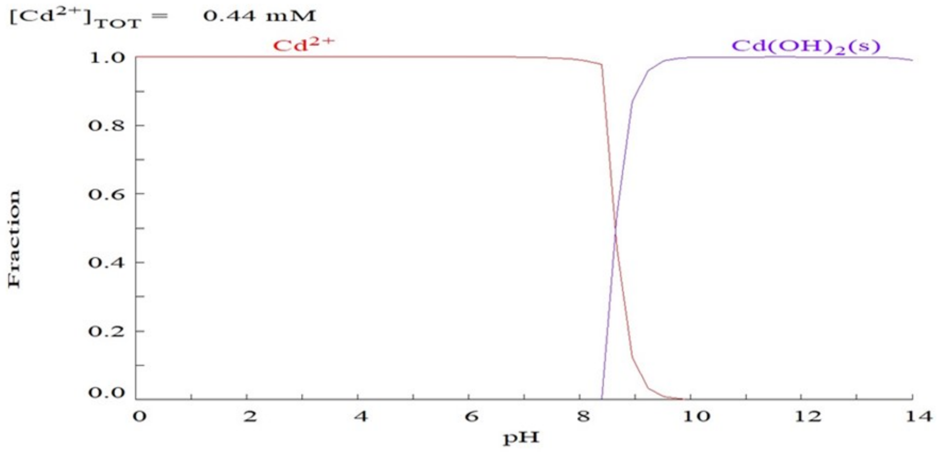

Figure S3: *Expected aqueous speciation of metal ion concentration (50 mg L^{-1}) for cadmium as a function of pH using Medusa/Hydra program.*

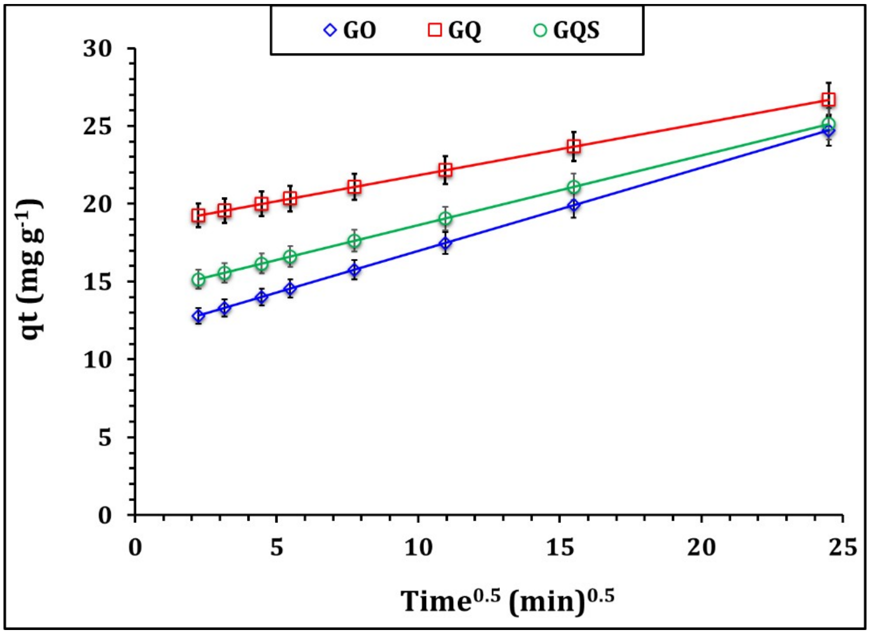

Figure S4: *Intraparticle diffusion (IPD) model plots for Cd(II) adsorption. Experimental conditions: initial ion concentration = 50 mg L⁻¹, pH = 5.01, dose = 0.5 g L⁻¹, temperature = 25°C.*

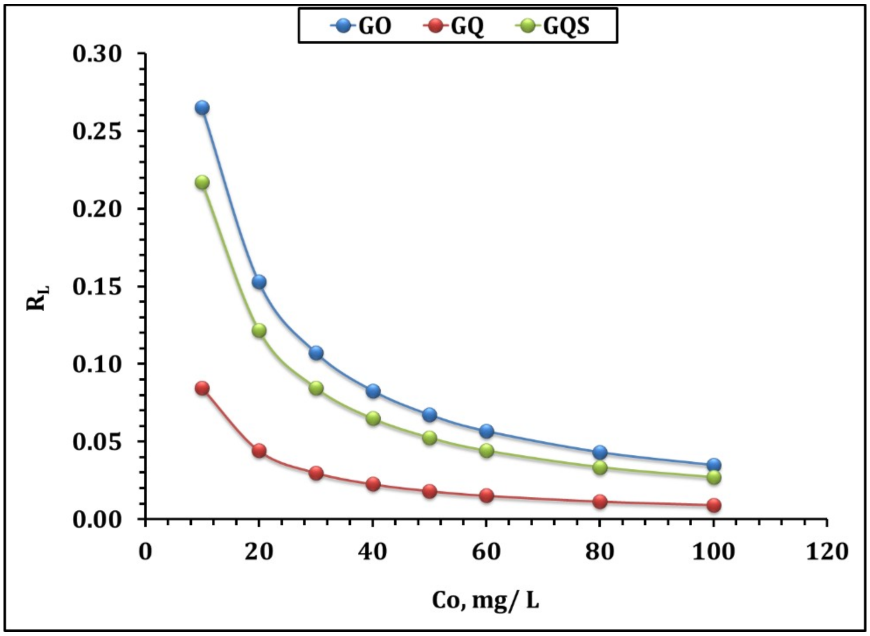

Figure S5: *Separation factor (R_l) for Cd(II) adsorption process using* GO, GQ, GQS sorbents.

Table S1: *Kinetic, isotherm, and thermodynamics equations for and Cd(II) sorption process* ***[1-7]***.

Kinetics	Equations
**Pseudo-first-order**	$q_t = q_1\left(1 - e^{-k1t}\right)$
**Pseudo-second-order**	$q_t = \frac{1}{\left(1 \mid k_2q_2^2\right) + \left(t \mid q_2\right)}$
**Intra-particle diffusion model (IPD)**	$q_t = K_{id}t^{0.5} + C_i$
**Isotherms**	**Equations**
**Langmuir model**	$q_e = \frac{q_m k_L C_e}{1 + k_L C_e}$
**Freundlich model**	$q_e = K_F C_e^{1/n_F}$
**Temkin model**	$q_e = \frac{RT}{b_T}\ln K_T C_e$
**Sips model**	$q_e = \frac{q_S\left(k_S C_e\right)^{mS}}{1 + \left(k_S C_e\right)^{mS}}$
**Thermodynamics**	**Equations**
$\log K_C =- \frac{\Delta H^o}{2.303\,R} X \frac{1}{T} + C$	
$-\Delta G^o = 2.303\,RT\log K_C$	
$\Delta G^o = \Delta H^o - T\,\Delta S^o$	
**Fitting**	**Equations**
**Coordination coefficient (R^2)**	$R^2 = 1 - \frac{\sum_1^n \left(q_{exp} - q_{pred}\right)^2}{\sum_1^n \left(q_{exp} - \bar{q}_{exp}\right)^2}$
**Average relative error (ARE)**	$ARE = \frac{100}{n}\sum_1^n \frac{\left|q_{exp} - q_{pred}\right|}{q_{exp}}$

qe (mg g^{-1}) is the equilibrium concentration of metal ions species, and qt (mg g^{-1}) is the adsorbed amount of metal ions species ions after time t (min), Ce (mg L^{-1}) is equilibrium concentration of metal ions species. k_1 (min^{-1}) and k_2 (min^{-1}) are the rate constants for the pseudo first and second order, respectively. K_{id} (mg/g. $min^{0.5}$) is a rate constant, and C is the thickness of the boundary layer. q_m and q_S are the maximum sorption capacity (mg. g^{-1}) of Langmuir and Sips models. k_L (L. mg^{-1}), K_F (L/ mg), K_T (L min^{-1}), and K_S (L/ mg) are represent the constants of Langmuir, Freundlich, Temkin, and Sips models. n refer to the sorption intensity, b_T is Temkin constant that refers to the adsorption heat, mS is Sips constant. $\boldsymbol{q_s}$ is the theoretical isotherm saturation capacity (mg/g). K_C is a non-dimensional equilibrium constant and it equals K_d X 1000 X ρ **[4-5]**; T is the temperature (K), $\boldsymbol{R}$ is the universal gas constant (8.314 J mol $^{-1}$. K^{-1}), ρ is solution density g/ L, and C is a constant. R^2 and x^2 are the coordination and Chi-square coefficients respectively, the number of test points is n, the experimental equilibrium capacity is q_{exp} (mg g^{-1}), while the predicted capacity is q_{pred} (mg g^{-1}).

References:

1. Hu Q, Pang S, Wang D (2022) In-depth insights into mathematical characteristics, selection criteria and common mistakes of adsorption kinetic models: A critical review. Sep Purif Rev. 51(3):281–299.
2. González-López ME, Laureano-Anzaldo CM, Pérez-Fonseca AA, Arellano M, Robledo-Ortíz JR (2022) A critical overview of adsorption models linearization: methodological and statistical inconsistencies. Sep Purif Rev. 51(3):358–372.
3. Taha MH (2021) Sorption of U(VI), Mn(II), Cu(II), Zn(II), and Cd(II) from multi-component phosphoric acid solutions using MARATHON C resin. Environ Sci Pollut Res. 28(10):12475–12489.
4. Chen X, Hossain MF, Duan C, Lu J, Tsang YF, Islam MS, Zhou Y (2022) Isotherm models for adsorption of heavy metals from water—a review. Chemosphere. 307:135545.
5. Majd MM, Kordzadeh-Kermani V, Ghalandari V, Askari A, Sillanpää M (2022) Adsorption isotherm models: A comprehensive and systematic review (2010–2020). Sci Total Environ. 812:151334.
6. Masoud, A.M., Ammar, H., Elzoghby, A.A., El Agamy, H.H. and Taha, M.H., 2025. Rare earth elements adsorption from phosphoric acid solution using dendrimer modified silica gel as well as kinetic, isotherm, and thermodynamic studies. Journal of Rare Earths, 43(7), pp.1502-1512.
7. Ebelegi AN, Ayawei N, Wankasi D (2020) Interpretation of adsorption thermodynamics and kinetics. Open J Phys Chem. 10(3):166–182.

Table S2: *The relationship between Cd(II) adsorption capacity and* sorbent *dose under the following conditions: room temperature, a pH of 6.0, a starting concentration of 50 mg L^{-1}, reaction time of 240 min.*

Sorbent dose, g/ L	Sorption capacity, mg/ g		
	GO	GQ	GQS
0.3	39.3	78.8	65.5
0.5	36.2	58.2	48.2
1.0	30.9	36.4	33.5
2.0	20.0	24.3	21.2
3.0	14.3	16.5	15.5

Table S3: *The values of Morris-Weber model parameters.*

		GO	GQ	GQS
**Weber and Morris model**	**k_i (mg/g min$^{1/2}$)**	0.54	0.33	0.45
	**C**	11.6	18.5	14.1
	**R^2**	0.98	0.98	0.98

Table S4: *Cd(II) desorption from loaded sorbent using different solutions (2.0 g/L, room temperature; 120 min).*

Eluent type	Sorption efficiency, %
1.0 M Hydrochloric acid	98.1
1.0 M Sulfuric acid	76.7
1.0 M Nitric	64.3

Table S5: *Initial concentrations of constituents in raffinate wastewater, along with calculated removal efficiency (%), and distribution coefficient (kd) after treatment with* GQ sorbent.

**Parameter**	**Initial concentration, mg/ L**	**Removal efficiency, %**	**distribution coefficient, Kd**
Cd(II)	120.0	94.2	8.07
V(V)	60.0	20.0	0.13
Cr(III)	80.0	11.3	0.06
Si(IV)	300.0	23.3	0.15
Ca(II)	504.0	19.8	0.12
Na(I)	320.0	15.0	0.09
Cl-	800.0	8.8	0.05
